# Niche breadth explains the range size of European‐centred butterflies, but dispersal ability does not

**DOI:** 10.1111/geb.13717

**Published:** 2023-06-13

**Authors:** Johannes Hausharter, Sonia Rashid, Johannes Wessely, Patrick Strutzenberger, Dietmar Moser, Andreas Gattringer, Konrad Fiedler, Karl Hülber, Stefan Dullinger

**Affiliations:** ^1^ Department of Botany and Biodiversity Research University of Vienna Vienna Austria; ^2^ Vienna Doctoral School of Ecology and Evolution (VDSEE) University of Vienna Vienna Austria; ^3^ Department of Biological Diversity and Nature Conservation Environment Agency Austria Vienna Austria

**Keywords:** dispersal, European‐centred butterfly species, Lepidoptera, niche breadth, phylogenetically informed regressions, range size

## Abstract

**Aim:**

The breadth of ecological niches and dispersal abilities have long been discussed as important determinants of species' range sizes. However, studies directly comparing the relative effects of both factors are rare, taxonomically biased and revealed inconsistent results.

**Location:**

Europe.

**Time Period:**

Cenozoic.

**Major Taxa:**

Butterflies, Lepidoptera.

**Methods:**

We relate climate, diet and habitat niche breadth and two indicators of dispersal ability, wingspan and a dispersal tendency index, to the global range size of 369 European‐centred butterfly species. The relative effects of these five predictors and their variation across the butterfly phylogeny were assessed by means of phylogenetic generalized least squares models and phylogenetically weighted regressions respectively.

**Results:**

Climate niche breadth was the most important single predictor, followed by habitat and diet niche breadth, while dispersal tendency and wingspan showed no relation to species' range size. All predictors together explained 59% of the variation in butterfly range size. However, the effects of each predictor varied considerably across families and genera.

**Main Conclusions:**

Range sizes of European‐centred butterflies are strongly correlated with ecological niche breadth but apparently independent of dispersal ability. The magnitude of range size–niche breadth relationships is not stationary across the phylogeny and is often negatively correlated across the different dimensions of the ecological niche. This variation limits the generalizability of range size–trait relationships across broad taxonomic groups.

## 1 INTRODUCTION

Geographical range size is a metric to measure the ecological “success” of a species (Faurby & Antonelli, 2018) and, complementarily, an important indicator of its risk of extinction in the Anthropocene (Purvis et al., 2000; Rodríguez et al., 2007). The determinants of range size have hence long intrigued biogeographers and ecologists (Brown et al., 1996; Wallace, 1876), and are, more recently, of renewed interest in conservation biology (Angert et al., 2011; Slatyer et al., 2013). Apart from historical factors like evolutionary age and region of speciation (Brown et al., 1996; Whittaker & Fernández‐Palacios, 2007), a number of ecological factors, which presumably interact with evolutionary processes (Dullinger et al., 2012; Slatyer et al., 2013), have been discussed. In particular, ecological theory predicts that the geographical distribution of species is a function of three components: its abiotic as well as its biotic requirements, and its ability to reach a site during its evolutionary lifetime (Soberón, 2007). As a consequence, two ecological features of species appear intuitively linked to range size.

First, broad ecological niches allow species to occupy large ranges (Carscadden et al., 2020; Slatyer et al., 2013). Species which are able to colonize multiple habitat types (Gaston, 1994), utilize a wide range of food resources (Quinn et al., 1997) or those physiologically adapted to tolerate varied climatic conditions (Bozinovic et al., 2011) should encounter suitable conditions across larger geographical areas more frequently than more specialized species. Consequently, these different dimensions of ecological niche breadth jointly determine the species' potential ranges. Second, species with high dispersal abilities are more likely to colonize suitable but hitherto unoccupied habitats, overcome geographic barriers (Brown et al., 1996) and ensure the persistence of populations in suboptimal habitats via continued propagule supply or recurrent immigration (Alzate et al., 2019). In contrast, strong dispersal limitation fosters high rates of speciation (Suárez et al., 2022), which in turn can lead to small range sizes (Webb & Gaston, 2000). However, individual dispersal and a species' range act at vastly different spatial scales (up to 1,000s of metres vs. up to 1,000s of kilometres), calling the importance of dispersal for determining range sizes into question. Nevertheless, dispersal certainly plays a role during early range expansion, for example, following a speciation event or after a rapid environmental change (e.g., post‐glacial re‐colonization), while its role might subsequently diminish over time (Lester et al., 2007).

The relationship between range size and niche breadth has been studied repeatedly for various taxa and at various spatial scales. In their meta‐analysis, Slatyer et al. (2013) concluded that such a relationship actually does exist, but with only moderate average effect size and considerable variation across taxonomic groups and niche dimensions. A comprehensive meta‐analysis on the effects of dispersal ability on range size is lacking so far, at least for terrestrial environments (see Lester et al., 2007, for the marine realm). However, a number of individual studies suggest that dispersal ability is positively related to range size in at least some taxonomic groups (e.g., Brändle et al., 2002; Ceolin & Giehl, 2017; Dullinger et al., 2012; Li et al., 2016; Luo et al., 2019; McCulloch et al., 2017) although this relationship does not appear to be universal (e.g., Alzate & Onstein, 2022; Lester et al., 2007; Varzinczak et al., 2020).

Interestingly, research strands on niche breadth–range size and dispersal–range size relationships have largely been separated so far. The few studies that have combined measures of niche breadth and indicators of dispersal ability in range size studies have been taxonomically biased towards terrestrial vertebrates, mostly mammals, and provided inconsistent results on the relative effects of the two factors (Faurby & Antonelli, 2018; Li et al., 2016; Varzinczak et al., 2020). Consequently, a more complete understanding of what determines range size is slow to emerge. Additional studies that directly compare range size effects of niche and dispersal traits should ideally cover yet understudied taxonomic groups and include different axes of the ecological niche as, for example, environmental tolerance and diet breadth are not necessarily correlated (Carscadden et al., 2020) and may relate differently to range size (Slatyer et al., 2013). Moreover, individual clades within a taxonomic group can exhibit different degrees of niche divergence, neutral evolution or conservatism (i.e., phylogenetic non‐stationarity; Diniz‐Filho et al., 2010). Thus, the strength and even the direction of relationships between species traits and range size may vary substantially between clades due to such evolutionary idiosyncrasies (Varzinczak et al., 2020). Considering phylogenetic non‐stationarity in range size–trait relationships can further improve our understanding of the determinants of range size.

In this study, we investigate niche breadth–range size and dispersal–range size relationships in European‐centred butterflies. Among invertebrates, the butterflies of Europe are a taxon particularly suited for studying these questions because we know both their distribution and traits better than those of most other insects. In addition, butterflies are closely bound to the thermal conditions of their environment (Dennis, 2020), and they show pronounced variation in larval host breadth (Altermatt & Pearse, 2011) as well as in dispersal ability, or at least dispersal behaviour (Dennis et al., 2013). We created global range maps of species by assembling maps from regional faunal monographs, together with occurrence records from an online database. Additionally, we compiled information on traits and habitat requirements of species to derive three measures of the ecological niche (climate niche breadth, diet niche breadth and habitat niche breadth) and two dispersal indicators (a rank‐based classification of dispersal tendency and wingspan). We then related these five predictors to the size of the species ranges in a phylogenetic analysis framework. In particular, we expect (1) the range sizes of butterfly species to be positively correlated with both their ecological niche breadth and their dispersal ability; and (2) range sizes to be more closely correlated with the three niche dimensions than to dispersal ability. Furthermore, we examined whether (3) the strength and direction of these correlations vary across the butterfly phylogeny.

## 2 METHODS

### 2.1 Study system

The European butterfly fauna comprises 496 species (Wiemers et al., 2018). Here, we excluded from the total set: (1) species which have their main area of distribution in Africa, Anatolia or Asia and just reach the eastern‐ or south‐eastern‐most margins of Europe; (2) species whose European distribution is restricted to Macaronesia, which politically belongs to Europe, but not biogeographically; (3) seasonal/rare migrants which are unable to establish permanent populations in Europe and (4) neobiota. In general, we followed an inclusive approach to species delimitation and pooled taxa whose taxonomic status (separate species vs. subspecies) is debated. Consequently, we included 374 butterfly species in our analyses (Supporting Information Table S1).

### 2.2 Range size

To obtain global range sizes of these 374 species, we combined published distribution maps from faunal monographs (Supporting Information Table S2). As these maps refer to the species' distributions in the second half of the 20th century, we modified the range margins to consider more recent findings represented by occurrence records derived from an updated version of the database based on Kudrna et al. (2011) and from the Global Biodiversity Information Facility (GBIF; https://www.gbif.org/) database (GBIF, 2020a, 2020b). Data for *Hipparchia blachieri* were downloaded manually because the automatic download failed to provide data for this species. Published regional distribution maps were digitized, geo‐referenced and merged using ArcGIS (Environmental Systems Research Institute [ESRI], 2020) to obtain polygons representing the species' geographical ranges. We cleaned occurrence records using the “CoordinateCleaner” package (Zizka et al., 2019) in R (R Core Team, 2021) to remove duplicate and zero longitude or latitude coordinates as well as coordinates corresponding to biodiversity institutions, a country's capital or its centroid. We further removed records with spatial uncertainties of coordinates larger than 10 km or those older than 1980. The latter limit was set (1) because we considered the distribution pre‐1980 as well covered by the range maps; and (2) to match the timespan covered by the climate data (see “Climate niche breadth” below). The remaining records were checked manually for errors based on outdated nomenclature, obvious misidentifications, widely extralimital records and recently discovered cryptic species diversity not (fully) covered by GBIF. The cleaned records were then aggregated to a 5‐arc‐minute grid and used to modify the polygons of the digitized distribution maps or supplement them in regions where such maps were not available. As a result, the range sizes of 246 (66%) species changed by more than ±20% compared to the ranges published in the faunal monographs. Range sizes increased by more than 100% for 63 species, and decreased by more than 50% for 7 species. The species' range sizes were then calculated as the geographical area (km^2^) spanned by their range polygons. As the range maps are necessarily coarse and some range change may have occurred since their publication (the contemporary cut‐off date for occurrence records was June 2020), this workflow may have resulted in inaccuracies at range margins. However, given the huge variation in range size across the 374 species, we do not expect these inaccuracies to bias our results.

### 2.3 Climate niche breadth

We calculated the species' climate niches using 19 bioclimatic variables downloaded from the CHELSA (Climatologies at high resolution for the earth's land surface areas) database version 1.2 (Karger et al., 2017a, 2017b). These variables are provided for the years 1979 to 2013 at a 30‐arc‐second resolution and were aggregated to a 5‐arc‐minute grid using the area‐weighted mean. We then calculated a principal component analysis (PCA) based on 10,000 cells randomly selected from the global grid (PCA loadings are depicted in Supporting Information Table S3) and predicted principal component scores for the remaining cells. For each species, we extracted the first four principal component scores of all cells containing GBIF records (cleaned records within their ranges as defined above). From these PCA scores, we calculated the species' climate niches by constructing hypervolumes via one‐class support vector machine learning models using the function “hypervolume” of the “hypervolume” package (Blonder et al., 2014). For 30 species, 25 of which are local endemics, the hypervolume could not be calculated because GBIF records extend over less than five 5‐arc‐minute cells. To avoid biasing our results by excluding strongly range‐restricted species, we assigned these 25 endemic species the minimum hypervolume calculated for any of the remaining species (also a local endemic). The 5 non‐endemic species with insufficient occurrences for hypervolume calculations were discarded from further analyses, thus reducing the total number of species to 369.

The number of 5‐arc‐minute cells occupied varied strongly between species (reaching up to 24,077 cells). Because sample size (i.e., number of cells) might affect the size of the calculated niche volume, we computed an additional set of hypervolumes (i.e., climate niche breadths) by randomly selecting a maximum of 50 cells per species (i.e., the hypervolume of those 70 species with less than 51 cells remained unchanged). This selection was repeated 10 times. The robustness of the chosen approach as well as alternatives are discussed in the Supporting Information “Sample size and climate niche breadth”.

### 2.4 Diet niche breadth

We used the phylogenetic diversity of host plant genera utilized by butterfly larvae as a measure to quantify diet niche breadth. Phylogenetic rather than taxonomic diversity (i.e., simple counts of host plant genera) was used to estimate diet niche breadth to better account for the phylogenetic relatedness (and therefore often phytochemical similarity) among host plants (Symons & Beccaloni, 1999). We compiled 2826 data records on larval host plants from the available literature and online sources (Supporting Information Table S2). Phylogenetic relationships between hosts were derived from a recently published species‐level meta‐tree of seed plants (Smith & Brown, 2018), which was constructed from GenBank and Open Tree of Life taxa and a taxonomic backbone provided by Magallón et al. (2015). As information on host plants was frequently given only at the genus level, we pruned the tree to contain only the 379 plant genera utilized by at least 1 of the 369 study species. The phylogenetic diversity of host plant genera was then determined as Faith's PD (Faith, 1992) using the function “pd” of the package “picante” (Kembel et al., 2010).

### 2.5 Habitat niche breadth

We collected data on the reproductive habitats of the butterflies from literature (Supporting Information Table S2). Based on this information, we defined seven broad habitat types: wetlands, sparsely vegetated land, grasslands, shrubland, forest, mixed cultivated land and arable land. A species' habitat niche breadth was then determined as the number of these habitat types used for reproduction.

### 2.6 Dispersal tendency

We classified the butterflies' dispersal tendency as sedentary (269 species), moderately mobile (89 species) and dispersive (11 species) based on the assessments made by Bink (1992) and Settele et al. (1999). Thereby, we merged Bink's original, expert assessment‐based nine‐level categorization into three classes (sedentary, moderately mobile and dispersive) to minimize the effect of potential miscategorizations and added species not already classified based on our own assessment.

### 2.7 Wingspan

Wingspan was evaluated as a moderately satisfactory proxy of dispersal ability in Lepidoptera, especially with larger numbers of diverse species (Sekar, 2012; Slade et al., 2013). As opposed to dispersal tendency, which also accounts for behavioural aspects, wingspan is a purely physical correlate of dispersal ability, we considered the two variables to be complementary rather than redundant. We acquired wingspan data from the literature (including field and lab measurements; Supporting Information Table S2) in the form of minimum and maximum values (mm). For the colonization of new habitats, only the size of female butterflies is relevant. We, hence, focused on values explicitly given for females (126 species) and used those given indiscriminately for both sexes for the remaining butterflies (243 species). We calculated the geometric mean of the minimum and maximum for each species and study and averaged these values for each species (i.e., across studies).

### 2.8 Butterfly phylogeny

We assembled sequence data of one mitochondrial and seven nuclear genes, cytochrome c oxidase subunit I (COI), wingless (WGL), elongation factor 1‐alpha (EF1A), ribosomal protein S5 (RPS5), glyceraldehyde 3‐phosphate dehydrogenase (GAPDH), carbamoyl‐phosphate synthetase 2 (CAD) and isocitrate dehydrogenase (IDH). Completeness of sequence data for each gene (i.e., percentage of species for which sequence data were available) was as follows, COI 100%, WGL 59%, EF1A 54%, RPS5 28%, GAPDH 27%, CAD 20% and IDH 11%. See Supporting Information Table S4 for a list of BOLD process IDs and NCBI GenBank accessions. Sequences of individual genes were aligned using the online version of MAFFT (Katoh & Standley, 2013) on auto settings. The combined aligned length of all genes was 5156 bp. The best‐fitting partitioning scheme was determined with Partitionfinder v1.1.1 (Lanfear et al., 2012). Mitochondrial and nuclear genes were treated as exclusive entities in Partitionfinder runs and the search algorithm was set to “greedy”. Model selection was performed using the Bayesian information criterion (BIC). The best‐fitting scheme partitioned the data into 11 partitions (see Supporting Information Table S5 for details). Estimation of phylogenies was done in BEAST v2.6.1 (Bouckaert et al., 2019). All site models were unlinked, and the best‐fitting partitioning scheme as determined by Partitionfinder was applied in BEAUTi. A separate log‐normal relaxed clock model was applied to each partition and the tree prior was set to the Yule model. Results from Chazot et al. (2019), referred to as “core analysis” in Chazot et al. (2019), were used for time calibration (see Supporting Information Table S6 for calibration points and prior settings). Accordingly, we used six calibration points corresponding to the root node of the five butterfly families and the root of the entire phylogenetic tree. All time‐calibrated clades were constrained to be monophyletic. Additionally, we constrained the relationships between the five butterfly families (Hesperiidae, Lycaenidae, Nymphalidae, Papilionidae and Pieridae) to the widely accepted and well‐supported branching pattern recovered in all phylogenetic studies on butterflies since Wahlberg et al. (2013). The BEAST run was performed with a chain length of 1.1 × 10^8^ with trees being sampled every 10,000^th^ generation. The BEAGLE library (Ayres et al., 2012) was used for parallelization. Log files were examined with Tracer v1.7 (Rambaut et al., 2018) to ensure sufficient effective sample sizes for all parameters (>200). The tree sample was summarized using TreeAnnotator (Drummond & Rambaut, 2007) where the first 10^7^ states were removed as burn‐in. Finally, common ancestor heights were annotated to the maximum clade credibility tree.

### 2.9 Analyses

To investigate the role of the three measures of niche breadth (climate, diet and habitat) and the two dispersal indicators (dispersal tendency and wingspan) in shaping range sizes of butterflies, we used phylogenetic generalized least squares (PGLS) models. These models account for phylogenetic relatedness among species by estimating the expected covariance structure of the model residuals and modifying intercept and slope estimates accordingly (Symonds & Blomberg, 2014). PGLS models were calculated using the “gls” function of the “nlme” package (Pinheiro et al., 2021). We log‐transformed the response variable (i.e., range size) and square root transformed all continuous predictor variables (i.e., climate niche breadth, diet niche breadth, habitat niche breadth and wingspan). These predictor variables were further standardized to zero mean and unit standard deviation. Before calculating the models, we also checked the correlation structure of predictor variables to avoid high collinearity (*r*/*ρ* ≥ 0.7).

We first calculated two full models including all predictor variables (i.e., climate niche breadth, diet niche breadth, habitat niche breadth, dispersal tendency and wingspan), one based on a Brownian motion (BM) and one based on the Ornstein–Uhlenbeck (OU) model of evolution for estimating the covariance structure. The covariance structures were calculated using the “corBrownian” and “corMartins” functions of the “ape” package (Paradis & Schliep, 2019). A likelihood ratio test revealed a better fit of the OU model (*Χ* ^2^(1) = 312.2, *p* < 0.001) which was, consequently, used for further analyses.

We then compared the full PGLS model to five reduced models, each fitted by dropping one of the predictors. The alpha parameter of the OU model of evolution was held constant (i.e., at the value estimated for the full model). To quantify the importance of individual variables in explaining species' range sizes, we calculated the partial pseudo‐R^2^ proposed by Ives (2019; R^2^_pred_) using the “R2_pred” function of the “rr2” package (Ives & Li, 2018). This pseudo‐R^2^ is based on the variance in the difference between observed and predicted data. The full PGLS model was further compared to a full OLS model (i.e., an ordinary least squares regression including all predictors) to obtain a partial *R* ^2^ value for the phylogeny. The total *R* ^2^ of the full PGLS model was calculated by comparing it to an OLS null model (i.e., a model without predictors).

To evaluate whether differences in the number of occurrence records among species affect the importance of the climate niche breadth variable, PGLS analyses were repeated using the 10 replicates of the climate niche breadth variable as calculated from a maximum of 50 occupied cells per species. These models are hereafter termed “constrained” in contrast to the “unconstrained” models which use the climate niche breadth based on the full set of occupied cells.

Finally, to explore how relationships between range size and the predictors vary across the butterfly phylogeny, we further calculated a multivariate, phylogenetically weighted regression (PWR; Davies et al., 2019). Thereby, a number of weighted regressions equal to the number of species are calculated. For each regression, another species is defined as the focal species with other species weighted according to their phylogenetic distance from the focal species. The applied distance weighting function may contain a parameter determining the decline in weights with phylogenetic distance. We utilized a distance weighting function based on an OU model of trait evolution and determined this parameter using the “subplex” optimization function. An OU covariance matrix is then calculated and used as weights in the weighted regressions. The estimates obtained by these weighted regressions are then interpreted as the effects of predictors on the range size of the respective focal species. We calculated the PWR model by utilizing the functions provided by Davies et al. (2019).

## 3 RESULTS

**FIGURE 1:**
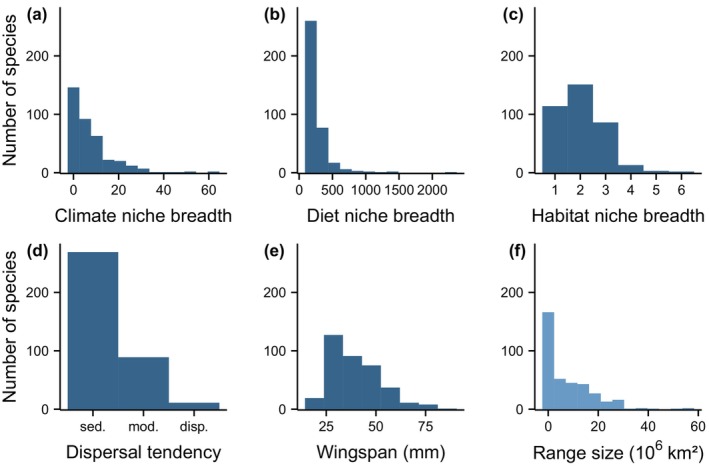
Frequency distributions of the five predictor variables describing niche breadths (a–c) and dispersal ability indicators (d–e), as well as the range sizes (f) of 369 European‐centred butterfly species. Climate niche breadth, diet niche breadth and habitat niche breadth represent hypervolumes derived from a PCA based on 19 bioclimatic variables, the phylogenetic diversity of host plant genera and the number of broad habitat types used for reproduction respectively. Dispersal tendency (sed., sedentary; mod., moderately mobile; disp., dispersive) was determined by expert assessment, based on Bink (1992) and Settele et al. (1999).

Global ranges of European‐centred butterflies varied by 4 orders of magnitude (Supporting Information Table S1). The set of 369 species (Papilionidae: 13 spp., Pieridae: 40 spp., Nymphalidae: 179 spp., Lycaenidae: 100 spp. and Hesperiidae: 37 spp.) included local endemics of small mountain ranges (e.g., *Hipparchia leighebi*, the species with the smallest range size: 1.2 × 10^2^ km^2^) as well as species found across all the temperate and boreal Northern hemisphere (e.g., *Papilio machaon*, the species with the largest range size: 5.6 × 10^7^ km^2^). Range sizes varied considerably even within genera, for example, by 2, 3 and 4 orders of magnitude in *Colias, Erebia* and *Polyommatus* respectively. However, most species had comparatively small ranges (Figure 1f).

The distribution of the five predictor variables (niche breadth measures and dispersal indicators) was also highly skewed towards small values (Figure 1a–e). The number of host plant genera utilized by the individual species ranged from 1 to 34 (mean = 4.4), with most species (35%) representing diet specialists feeding on only one plant genus. The number of reproductive habitats per species ranged from 1 to 6 (mean = 2), with most species (41%) being able to reproduce in two habitat types. Correlations among predictor variables were low (Supporting Information Table S7).

The unconstrained PGLS model explained approximately 59% of the range size variation in European‐centred butterflies (Table 1; for a visualization of univariate relations in the raw data, see Supporting Information Figure S1). Two of the niche breadth dimensions were significantly and positively related to range size (Table 2). By far, the most important single predictor was climate niche breadth with 38% of the variation uniquely attributed to this predictor. Habitat niche breadth accounted for only minor fractions of the variation in the unconstrained PGLS model, while still exhibiting a significant effect on range size. In contrast, diet niche breadth and both indicators of dispersal ability (i.e., dispersal tendency and wingspan) did not significantly affect range size. Seven per cent of the variation in range size was attributed to the phylogeny.

**TABLE 1:** Importance of predictor variables in explaining range sizes of 369 European‐centred butterfly species derived from PGLS models.

Model component	Unconstrained	Constrained
	*R* ^2^	*R* ^2^
Full model	0.59	0.48 (0.46–0.49)
Climate niche breadth	0.38	0.22 (0.18–0.24)
Diet niche breadth	0.00	0.04 (0.03–0.05)
Habitat niche breadth	0.04	0.07 (0.07–0.08)
Dispersal tendency	0.00	0.00 (0.00–0.00)
Wingspan	0.00	0.00 (0.00–0.00)
Phylogeny	0.07	0.07 (0.06–0.09)

*Note*: Values represent the partial *R* ^2^ values derived from comparisons of a full model including all predictors to a set of models lacking the respective predictor. Values for the phylogeny were obtained by comparing the full model to an equivalent OLS full model. The value for the full PGLS model represents the total *R* ^2^ value. For the unconstrained models, climate niche breadth was calculated based on the full set of occupied cells, while the replicates of the climate niche breadth for the 10 replicated model fits of the constrained models were derived by randomly selecting a maximum of 50 occupied cells per species. Partial *R* ^2^ values for the constrained models are given as mean (min – max) across 10 replicated model fits.

**TABLE 2:** Summary of the full PGLS models relating three niche dimensions and two dispersal indicators to the range sizes of 369 European‐centred butterfly species.

Model component	Unconstrained			Constrained		
	Estimate ± *SE*	*t*‐value	*p*‐value	Estimate ± *SE*	*t*‐value	*p*‐value
Climate niche breadth	1.56 ± 0.10	15.53	**<0.001**	1.08 ± 0.11	10.14	**<0.001**
Diet niche breadth	0.00 ± 0.09	0.03	0.980	0.36 ± 0.10	3.49	**0.001**
Habitat niche breadth	0.31 ± 0.09	3.38	**0.001**	0.51 ± 0.10	4.88	**<0.001**
Dispersal tendency	−0.07 ± 0.26	−0.28	0.780	0.25 ± 0.30	0.82	0.412
Wingspan	−0.06 ± 0.09	−0.70	0.484	0.02 ± 0.10	0.22	0.825

*Note*: Estimates for the constrained models represent the means over these 10 model fits. Corresponding standard errors were averaged according to Burnham and Anderson (2004; equation 4). The average *p*‐values for the 10 replicated model fits were then calculated based on *t*‐values derived from these averaged estimates and standard errors. Significant effects are shown in bold.

Abbreviation: *SE*, standard error.

The results of the constrained PGLS models were qualitatively largely in accordance with the unconstrained PGLS model (Tables 1 and 2). However, the average total variation explained across the 10 randomizations was only 48%. Climate niche breadth remained the most important predictor but explained less of the variance (22%) than in the unconstrained PGLS model, while the amount of variance explained by the highly significant habitat niche breadth variable increased (7%). In contrast to the unconstrained model, diet niche breadth was a significant predictor for range size in these models, even though it accounted for only 4% of the variation in range size. Variables indicating dispersal ability, averaged across all 10 replicated model fits, remained without a significant effect on range size. Seven per cent of the variation continued to be attributed to the phylogeny.

**FIGURE 2:**
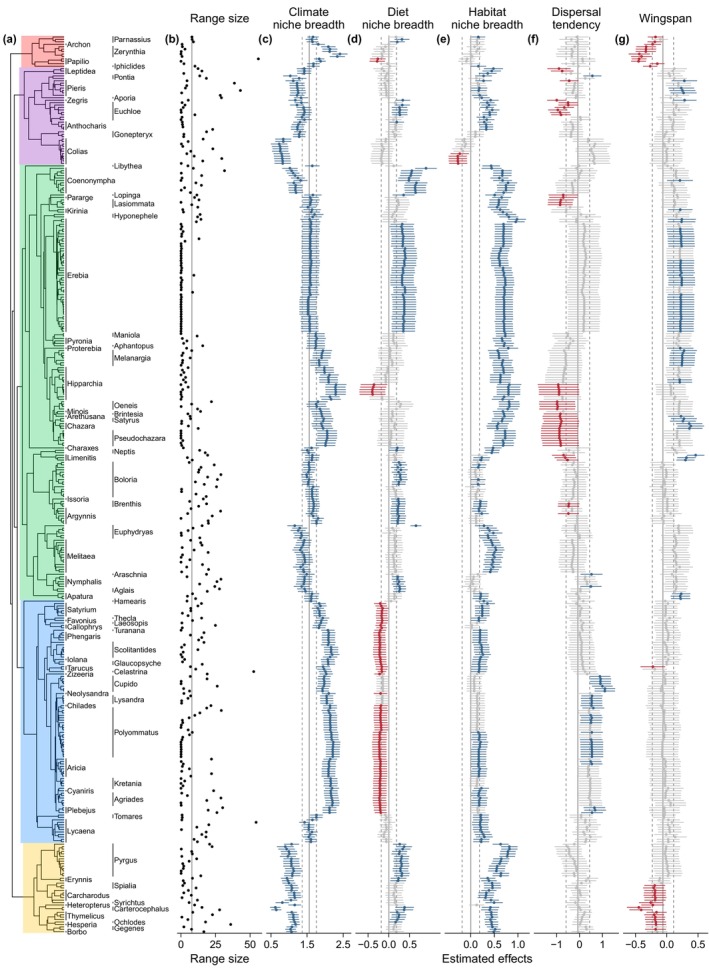
Species‐specific effects of five predictor variables (c–g) on range sizes (b; in 10^6^ km^2^) of 369 butterfly species derived from a multivariate phylogenetically weighted regression. Phylogenetic relationships (a) were derived from sequence data of one mitochondrial and seven nuclear genes. Coloured highlights correspond to butterfly families (red = Papilionidae, purple = Pieridae, green = Nymphalidae, blue = Lycaenidae and yellow = Hesperiidae). Predictors include three niche dimensions (c–e) and two indicators of the dispersal ability of species (f–g). Dots and error bars represent the estimated marginal coefficients (i.e., slopes) and their 95% confidence interval respectively. Greyed‐out bars indicate non‐significant effects of predictors (i.e., the 95% confidence intervals overlap with zero), while blue and red bars represent significantly positive and negative effects respectively. The vertical line in the range size column (b) represents the mean range size across all species. Vertical solid and dashed lines in the predictor columns (c–g) represent PGLS estimates of the unconstrained model and their 95% confidence intervals respectively.

In the PWR model, the effects of climate niche breadth on range size were positive for all species (Figure 2c) while diet niche breadth had no significant effects on the range sizes of almost half of the species (166 [45%]) and significant positive and negative effects for 120 (33%) and 83 (22%) species respectively (Figure 2d). Habitat niche breadth had significant positive effects on the range sizes of most species (280 [76%]; Figure 2e) and significantly negative effects for only five (1%) species. The strength of these effects varied considerably across the butterfly phylogeny. This is especially true for the estimated effects of climate and diet niche breadth, which were negatively correlated (*r* = −0.59, *p* < 0.001). Phylogenetic variation in niche breadth–range size relations markedly clustered at the genus level. In the species of the genus *Coenonympha*, climate niche breadth showed lower‐than‐average effects on their range size, while both diet and habitat niche breadth had higher‐than‐average effects. A similar, although less pronounced pattern emerged for the genus *Pyrgus*. In the genus *Polyommatus*, on the other hand, climate niche breadth had very strong positive effects on range size, while the effects of diet niche breadth were negative.

In contrast to niche dimensions, dispersal tendency and wingspan affected the range size of only 78 (21%) and 97 (26%) species respectively (Figure 2f,g). These effects were inconsistent in their direction, with 34 versus 44 species showing positive versus negative effects of dispersal tendency, and 66 versus 31 species showing positive and negative effects of wingspan on range size respectively.

## 4 DISCUSSION

Our results demonstrate that up to 59% of the variation in the range sizes of European‐centred butterflies can be explained by five variables describing their ecological niche breadth and dispersal ability. This is remarkable, given that many additional variables which potentially determine a species' distribution and, hence, its range size—such as biotic interactions other than host plant relations (Wisz et al., 2013), historical contingencies like palaeo‐climatic fluctuations (Schmitt et al., 2006) or various life history traits—have not been accounted for. In contrast to our expectations, the relations of range size to niche breadth metrics and dispersal ability indicators were highly uneven. While niche breadth explains a considerable part of butterfly range size, the effect of dispersal ability turned out to be negligible. However, relationships between range size and the predictors show considerable phylogenetic structure, suggesting that the strategies or attributes that help species to colonize large ranges are to a certain extent clade specific.

### 4.1 Ecological niche as an important determinant of range size

Climate niche breadth is the single most important predictor of range size in the studied butterflies, corroborating the results of the most recent meta‐analysis on niche breadth–range size relationships in terrestrial species (Slatyer et al., 2013). However, its effect size strongly depends on the way climate niche breadth is calculated. Accounting for a possible sampling effect by capping the number of occurrences included in the climate niche breadth calculation distributes more of the explained variances towards the other two niche dimensions in the constrained models. However, such capping may underestimate the real climate niche breadth, especially of more widespread species, and could thus weaken its relation to range size. Yet, when calculated from an uncapped number of occurrences, the climate niche breadth may have been underestimated for species which are under‐represented in the GBIF data rather than for well‐sampled and often widespread species. Such a sampling effect could have potentially inflated both the strength of the relationship between climate niche breadth and range size, as well as the total variation explained by the unconstrained model. We hence argue that our constrained and unconstrained models likely represent upper and lower boundaries of the relationship between climate niche breadth and range size and that the actual effect size is intermediate between the results of these two models.

The dominant effect of a large climate niche likely reflects the fact that ectothermic organisms such as butterflies are highly sensitive to ambient temperatures (Dennis, 2020) and that temperature conditions vary substantially across the area colonized by European‐centred butterflies, from the Mediterranean south and subtropical areas of western Asia to northern Siberia and the southern fringes of the Arctic. In particular, butterflies need to develop specific strategies to cope with low temperatures which at least seasonally dominate in temperate and boreal Eurasia. The level to which species have evolved such strategies likely allows them to colonize these huge areas to a varied degree. In line with this assumption, various modelling studies have suggested a strong imprint of climate on the distribution of European butterflies (e.g., Hill et al., 2002; Schweiger et al., 2012). Concomitantly, re‐surveys and population monitoring have demonstrated pronounced pole‐ward range shifts of European butterflies in response to recent climate warming (e.g., Chen et al., 2011; Eskildsen et al., 2013; Parmesan et al., 1999). These range shifts also include dieback at trailing range margins (Thomas et al., 2006), indicating that species also vary in their tolerance to warm and/or dry conditions. With climatic constraints setting limits at both the cold and the warm/dry range margins, a broader climate niche should consequently allow them to colonize larger areas.

The studied butterfly species differ widely in their larval diet breadth, from species monophagous on one host plant species (e.g., *Araschnia levana* on *Urtica dioica*) to those nearly omnivorous (e.g., *Celastrina argiolus*, feeding on flowers and developing seeds of plants in 14 families). However, most species have a rather limited host repertoire (Braga et al., 2021) and, consequently, their range size is potentially limited by the distribution of their hosts (Araújo & Luoto, 2007). Indeed, a couple of national‐scale studies have corroborated the effect of diet niche breadth on butterfly distribution (e.g., Dennis et al., 2005; García‐Barros & Benito, 2010; Quinn et al., 1998). However, at the European scale, many butterflies occupy ranges smaller than those of their plant hosts. Limitation by fodder plants has thus been argued to be less important than climatic constraints for the majority of species at the continental scale (Brändle et al., 2002; Schweiger et al., 2012). This inference is in line with the predictor importance suggested by our analyses of the global range size of the 369 species studied. While it was a negligible predictor in the unconstrained model, the importance of diet niche breadth for predicting range size was higher in the constrained models where the explanatory power of climate niche breadth was lower. As diet and climate niche breadth are correlated to a certain extent, their relative effects on range size cannot be fully disentangled. This correlation probably reflects the fact that a larger climate niche allows a species to come in contact and adapt to a larger number of potential hosts. Although host plant use is a phylogenetically conserved trait among butterflies (Menken et al., 2010; Wilson et al., 2012), integration of novel host plants into the larval resource niche is a recurrent phenomenon (Nylin et al., 2014). Such changes in host use are often associated with range shifts of either the hosts or the butterflies (Nylin et al., 2014) and have also been reported in the context of climate‐driven recent northern range expansions of European butterfly species (Pateman et al., 2012). Vice versa, the range expansion of polyphagous species is less restricted by host plant availability and may thus enable successive evolutionary broadening of the climate niche.

The effect of habitat niche breadth is larger than the one of diet niche breadth in our models, corroborating earlier results on its relationship with butterfly range size (Brändle et al., 2002; Cowley, Thomas, Wilson, et al., 2001). However, habitat breadth is the niche dimension most difficult to quantify. The index we used was interpreted as a metric characterization of habitat niche breadth, although differences among the considered habitats are not “equally spaced”, that is, grasslands and wetlands differ less from one another in many habitat characteristics than croplands from forests. As a corollary, a certain habitat niche breadth value can indicate quite different levels of flexibility in habitat use and the calculated niche breadth will depend on how habitats are categorized. Furthermore, habitat niche breadth was derived by summing habitat categories based on expert assessments and is therefore not directly comparable to metric variables such as climate niche breadth, which are computed from available observation data. We are still confident that this approach is preferable to using observational records and land cover data to derive habitat niche breadth in a conceptually more similar manner to climate niche breadth. While climate data is spatially highly autocorrelated and varies only gradually, land cover data can vary drastically within short distances. Even minor spatial inaccuracies in both the observation data and the land cover maps can therefore result in incorrect associations of species with certain habitat types, while these inaccuracies will have only minor, if any, effects on a species' climate niche breadth. However, to test whether our results are robust to the approach chosen for calculating habitat niche breadth, we also calculated another version of the habitat niche breadth variable by intersecting GBIF occurrence records with land cover data (for details, see Supporting Information “Alternative habitat niche breadth”). Following this approach, most species' habitat niche breadths were substantially overestimated, while the results of the models remained qualitatively similar, with a reduced importance of habitat niche breadth for predicting range sizes. We, therefore, consider expert knowledge as the clearly superior way of reliably linking butterfly species to their preferred reproductive habitats and calculating habitat niche breadth. However, given the caveats discussed, we think that the relative importance of climatic and habitat niche breadth for butterfly range size cannot be derived from our results with certainty and consider this an issue in need of further exploration.

While our results demonstrate the relation between niche breadth and range size, they cannot elucidate the direction of causality in this relationship, that is, whether a broader niche is the cause or consequence of a larger range. In fact, niche breadth and range size may be collectively determined by eco‐evolutionary processes, potentially resulting in a positive‐feedback loop between increased niche breadth and range expansion (Lancaster, 2020, 2022). As a result, a species may be comprised of a set of more specialized populations, each, or at least part of them, with a significantly narrower niche than the species as a whole. For example, *Lycaena dispar* populations in North‐western Europe inhabit wetlands where they usually feed on *Rumex hydrolapathum*. Yet, populations in Southern and Central Europe are not strictly associated with a single host plant species and habitat type, and even colonize urban areas (Lai & Pullin, 2004; Strausz et al., 2012). Additionally, Singer and Parmesan (2021) found that recently colonized populations of the North American butterfly species *Euphydryas editha* temporarily increased their diet niche breadths; however, when these populations persisted, their diet niche breadth reverted back towards its ancestral state. As our knowledge of intraspecific, local (or even temporal) differentiation in host or habitat preferences is anecdotal at best, a robust assessment of its effects on our results is impossible. However, widespread species should be more strongly affected by this issue than narrow‐ranged species as their populations are likely more spatially separated and ecologically differentiated.

### 4.2 No effect of dispersal ability on range size

Studies investigating the effect of dispersal ability of butterflies on their range sizes report mixed results. Cowley, Thomas, Roy, et al. (2001) reported a positive effect of an expert‐based mobility score on range size, a result corroborated by Brändle et al. (2002). However, Brändle et al. (2002) further found wingspan to be weakly negatively related to range size. In other flying insects, such as Plecoptera in New Zealand, positive correlations have been reported (McCulloch et al., 2017). Our own results, however, do not provide evidence for an effect of dispersal ability on the range size of European‐centred butterflies. The lack of a relationship may be due to the relatively long timespan over which post‐glacial colonization of current ranges has taken place. Dispersal ability may have played a role during initial phases of the Holocene re‐colonization of temperate and boreal Eurasia, but even weak dispersers may have had sufficient time to colonize larger ranges over the last 10,000 climatically relatively stable years. Indeed, estimates of recent range shifts under climate change suggest that butterfly species have expanded their northern‐range margins in Great Britain by c. 1 km/year, on average (Chen et al., 2011). In Finland, many butterfly species showed a rapid northward expansion driven by climate change (Pöyry et al., 2009). Such a rapid migration rate should have allowed at least the average species to reach full‐range filling during its expansion from glacial refugia, if no other constraints or barriers were in place. In addition, populations at expanding range margins may show evolutionary changes in dispersal ability and wing morphology (Hill et al., 2011) compared to populations in more stationary parts of the range due to differences in selection pressures. Averaging traits across the entire species, instead of focusing on frontier populations, may hence obscure actually existing relations between traits such as wingspan and range size.

We emphasize, however, that analysing the relationship between range size and dispersal ability in butterflies is compromised by the difficulties in measuring dispersal ability in mobile, yet rather short‐lived animals. Wingspan emerged as the most versatile morphological predictor of butterfly dispersal ability in a comparative study (Sekar, 2012), and selection for longer wings appears correlated with range expansion of insects under climate change (Hill et al., 2011). Nevertheless, its ability to predict movement distances measured in mark–release–recapture (MRR) studies is limited (Sekar, 2012). Moreover, MRR studies for European butterflies are biased towards species of non‐forest habitats that form rather closed demes within meta‐populations. Large‐sized and vagile species like larger Nymphalinae, Limenitidinae, Apaturinae or Papilionidae are under‐represented in MRR studies conducted in Europe. Thus, the relation between movement distance and wingspan is largely unknown for these species. Expert‐based indices like our rank‐based dispersal tendency, on the other hand, can be confounded by expert assumptions of how various traits such as body size and flight period duration affect dispersal distances (Stevens et al., 2010). Furthermore, range expansion is probably the result of rare long‐distance dispersal events rather than the individual's dispersal ability or behaviour (Lester et al., 2007). We conclude that our results do not provide entirely conclusive evidence for the lack of a relationship between dispersal ability and range size in European‐centred butterflies. However, we are still confident that such a relation, if it exists, is clearly weaker than those of range size with niche breadth.

### 4.3 Phylogenetic variation in range–size predictor relationships

The relationships between range size on the one hand, and the ecological niche as well as the dispersal abilities of species on the other hand, are not stationary across the phylogenetic tree of butterflies, as Figure 2 shows marked clustering at the family, and partly at the genus level. It appears that the degree of phylogenetic conservativism in particular traits modulates the importance of different niche dimensions, and to a lesser extent also dispersal ability for shaping species' range sizes. To give some examples, species in the genus *Erebia*, which is largely confined to high altitudes or latitudes, show a particularly pronounced effect of habitat and diet niche breadth on range size, but only an average effect of climate niche breadth. This may be the result of a common adaptation to cold climates in this genus which could limit the importance of climate niche breadth for range expansion, with habitat and diet variability complementarily gaining importance. Indeed, the species differ in their portfolio of host plants from the Poaceae, Juncaceae and Cyperaceae families; and they vary from specialists of alpine grasslands to generalists of mountain environments, including various types of forests, shrublands and rock/scree vegetation in addition to grasslands. In the genus *Lycaena*, by contrast, all European species are bound to feed on plants in the Polygonaceae family, which likely explains the non‐existent effect of diet breadth on range size in this group. As another example, the lycaenid tribe *Polyommatini* stands out with a particularly strong effect of climate niche breadth and, indeed, the species in this group appear highly variable in their climate niches as they comprise Mediterranean endemics, an Arctic species (*Agriades quilo*), as well as substantially more widespread species. More generally, in groups in which the effect of one niche dimension on range size is, for whatever reason, limited, the correlation of range size with niche breadth along one of the other dimensions strengthens. This is also demonstrated by the correlation between the estimated effects of climate niche breadth and diet niche breadth in the PWR model, indicating that species with narrow climate niches can still reach large ranges via a broad spectrum of utilized host plants and vice versa. Nevertheless, we note that not all patterns detected by phylogenetically weighted regression are easily interpretable in this way. All species in the genus *Coenonympha*, for example, feed on graminoids (Poaceae and Cyperaceae), but diet niche breadth still has a stronger than average relevance for range size variation in this group. However, the generic inference from these phylogenetic patterns is that the importance of species traits for range size varies considerably even in a relatively narrow group such as the European butterflies. The variation unexplained by cross‐taxon range size models (e.g., Slatyer et al., 2013) may thus not only be due to important predictors missing in these models but also due to the pronounced variation in the importance these predictors have for the range sizes of different clades.

## 5 CONCLUSIONS

In summary, our results suggest that ecological niche breadth rather than dispersal ability explains the range sizes of European butterflies. However, our analyses cannot elucidate the direction of causality between niche breadth and range size, which may be tied through reciprocal feedback. The big difference in the relative importance of these factors indicates the robustness of our results despite inherent difficulties in quantifying the species' dispersal ability. The relationships we found are further not stationary across the butterfly phylogeny, with niche breadth and dispersal ability varying in size and direction of their estimated effects on range sizes in different clades. In various butterfly clades, individual niche dimensions revealed opposing relationships with range size. For example, in the Lycaenidae family, habitat and climate niche breadth are positively related to range size, whereas larval diet breadth came out almost invariably as negatively related to range size. One might speculate that in the European Lycaenidae, the widespread association with ants during their larval stages, as an additional niche dimension (Fiedler, 2021; Pierce & Dankowicz, 2022), contributes to the unexpected inverse relationship between diet breadth and geographical range size. Whatever the detailed reasons might be, these findings suggest the existence of clade‐specific trade‐offs between different factors that promote range size. These trade‐offs are hardly explored and merit further scrutiny.

## FUNDING INFORMATION

Austrian Science Fund FWF (P 31014).

## CONFLICT OF INTEREST STATEMENT

We declare no conflict of interest.

## BIOSKETCH

**Johannes Hausharter** is a doctoral student at the University of Vienna, where he currently studies the effects of climate change on alpine plant populations. He is also interested in animal ecology, especially insects, and has previously worked on tropical Odonata.

## Supporting information


**Data S1:** Supporting Information

## Data Availability

The data (i.e., range size, niche breadth and dispersal data) and R script necessary to reproduce the presented results, as well as the range maps and the utilized butterfly phylogeny, are publicly available online in a Dryad digital repository (https://doi.org/10.5061/dryad.n8pk0p30x).
