# Supplementary material for: Niche breadth explains the range size of European‐centred butterflies, but dispersal ability does not
Source: Glob Ecol Biogeogr. 2023 Jun 13;32(9):1535–48. doi: 10.1111/geb.13717 (PMC10946795; doi:10.1111/geb.13717)
Supplement: Supplementary file 1 — Data S1: Supporting Information [file GEB-32-1535-s001.docx]

Supporting Information

**Niche breadth explains the range size of European-centred butterflies, but dispersal ability does not**

**Table S1.** Table of European-centred butterfly species considered in this study, including their range sizes. Note that the range maps from which range sizes were derived are necessarily coarse, potentially resulting in inaccuracies at range margins. Hence, range sizes might be biased towards higher values, especially for small-ranged species.

| **Species** | **Range size (km²)** | **Species** | **Range size (km²)** |
| --- | --- | --- | --- |
| *Aglais io* | 19,042,928 | *Aricia agestis* | 7,164,962 |
| *Aglais urticae* | 27,862,789 | *Aricia anteros* | 1,690,644 |
| *Agriades aquilo* | 18,691,588 | *Aricia artaxerxes* | 22,118,688 |
| *Agriades glandon* | 163,561 | *Aricia cramera* | 1,004,325 |
| *Agriades optilete* | 28,931,557 | *Aricia eumedon* | 22,087,103 |
| *Agriades orbitulus* | 4,650,560 | *Aricia montensis* | 576,213 |
| *Agriades pyrenaica* | 560,341 | *Aricia morronensis* | 207,436 |
| *Agriades zullichi* | 9,869 | *Aricia nicias* | 6,097,120 |
| *Anthocharis cardamines* | 22,945,158 | *Boloria aquilonaris* | 18,278,747 |
| *Anthocharis damone* | 1,085,734 | *Boloria chariclea* | 16,865,246 |
| *Anthocharis euphenoides* | 965,367 | *Boloria dia* | 14,516,610 |
| *Anthocharis gruneri* | 1,410,396 | *Boloria eunomia* | 25,716,764 |
| *Apatura ilia* | 10,600,351 | *Boloria euphrosyne* | 19,303,078 |
| *Apatura iris* | 8,351,046 | *Boloria freija* | 26,396,545 |
| *Apatura metis* | 4,706,914 | *Boloria frigga* | 24,067,014 |
| *Aphantopus hyperantus* | 15,744,781 | *Boloria graeca* | 441,490 |
| *Aporia crataegi* | 29,293,407 | *Boloria improba* | 5,988,838 |
| *Araschnia levana* | 18,487,511 | *Boloria napaea* | 10,897,527 |
| *Archon apollinus* | 820,708 | *Boloria pales* | 457,637 |
| *Arethusana arethusa* | 6,587,767 | *Boloria polaris* | 13,139,414 |
| *Argynnis adippe* | 19,879,812 | *Boloria selene* | 28,903,324 |
| *Argynnis aglaja* | 22,622,974 | *Boloria thore* | 12,668,736 |
| *Argynnis elisa* | 32,877 | *Boloria titania* | 10,448,847 |
| *Argynnis laodice* | 4,867,208 | *Borbo borbonica* | 16,573,966 |
| *Argynnis niobe* | 19,695,325 | *Brenthis daphne* | 12,798,044 |
| *Argynnis pandora* | 7,312,093 | *Brenthis hecate* | 6,305,135 |
| *Argynnis paphia* | 28,669,422 | *Brenthis ino* | 18,531,008 |

**Table S1**. Continued.

| **Species** | **Range size (km²)** | **Species** | **Range size (km²)** |
| --- | --- | --- | --- |
| *Brintesia circe* | 5,032,245 | *Colias myrmidone* | 4,245,972 |
| *Callophrys avis* | 946,268 | *Colias palaeno* | 29,534,693 |
| *Callophrys rubi* | 24,933,053 | *Colias phicomone* | 335,757 |
| *Carcharodus alceae* | 11,915,519 | *Colias tyche* | 9,553,423 |
| *Carcharodus baeticus* | 778,020 | *Cupido alcetas* | 6,215,701 |
| *Carcharodus flocciferus* | 7,496,753 | *Cupido argiades* | 26,258,652 |
| *Carcharodus lavatherae* | 5,369,271 | *Cupido carswelli* | 93,924 |
| *Carcharodus orientalis* | 2,066,984 | *Cupido decoloratus* | 1,951,094 |
| *Carterocephalus palaemon* | 27,957,986 | *Cupido lorquinii* | 170,507 |
| *Carterocephalus silvicolus* | 15,208,239 | *Cupido minimus* | 18,881,183 |
| *Celastrina argiolus* | 52,642,719 | *Cupido osiris* | 6,742,458 |
| *Charaxes jasius* | 1,729,421 | *Cyaniris semiargus* | 23,639,602 |
| *Chazara briseis* | 12,480,102 | *Erebia aethiopella* | 26,640 |
| *Chazara persephone* | 5,367,107 | *Erebia aethiops* | 9,334,501 |
| *Chazara prieuri* | 438,995 | *Erebia alberganus* | 260,833 |
| *Chilades trochylus* | 21,702,579 | *Erebia arvernensis* | 130,106 |
| *Coenonympha arcania* | 6,801,512 | *Erebia calcaria* | 25,504 |
| *Coenonympha corinna* | 39,030 | *Erebia cassioides* | 110,758 |
| *Coenonympha dorus* | 767,284 | *Erebia christi* | 5,236 |
| *Coenonympha gardetta* | 203,113 | *Erebia claudina* | 16,640 |
| *Coenonympha glycerion* | 14,807,050 | *Erebia disa* | 11,146,950 |
| *Coenonympha hero* | 10,814,645 | *Erebia embla* | 14,470,976 |
| *Coenonympha leander* | 2,627,173 | *Erebia epiphron* | 781,333 |
| *Coenonympha oedippus* | 8,981,298 | *Erebia epistygne* | 164,133 |
| *Coenonympha pamphilus* | 14,978,834 | *Erebia eriphyle* | 101,972 |
| *Coenonympha rhodopensis* | 255,414 | *Erebia euryale* | 2,925,681 |
| *Coenonympha tullia* | 31,516,918 | *Erebia flavofasciata* | 19,711 |
| *Colias alfacariensis* | 5,197,377 | *Erebia gorge* | 431,824 |
| *Colias aurorina* | 1,326,502 | *Erebia gorgone* | 44,456 |
| *Colias caucasica* | 218,118 | *Erebia hispania* | 65,858 |
| *Colias chrysotheme* | 7,132,933 | *Erebia lefebvrei* | 73,145 |
| *Colias erate* | 23,070,167 | *Erebia ligea* | 13,049,978 |
| *Colias hecla* | 15,771,847 | *Erebia manto* | 614,203 |
| *Colias hyale* | 16,061,605 | *Erebia medusa* | 8,402,103 |

**Table S1**. Continued.

| **Species** | **Range size (km²)** | **Species** | **Range size (km²)** |
| --- | --- | --- | --- |
| *Erebia melampus* | 178,769 | *Euchloe simplonia* | 204,681 |
| *Erebia melas* * | 273,414 | *Euchloe tagis* | 819,086 |
| *Erebia meolans* | 662,977 | *Euphydryas aurinia* | 13,641,695 |
| *Erebia mnestra* | 120,941 | *Euphydryas cynthia* | 173,634 |
| *Erebia montana* | 141,189 | *Euphydryas desfontainii* | 351,695 |
| *Erebia neleus* | 132,570 | *Euphydryas iduna* | 5,172,413 |
| *Erebia neoridas* | 235,419 | *Euphydryas intermedia* | 8,734,731 |
| *Erebia nivalis* | 40,891 | *Euphydryas maturna* | 15,297,079 |
| *Erebia oeme* | 535,370 | *Favonius quercus* | 8,348,389 |
| *Erebia orientalis* | 29,147 | *Gegenes nostrodamus* | 7,670,613 |
| *Erebia ottomana* | 336,590 | *Gegenes pumilio* | 2,112,265 |
| *Erebia palarica* | 51,430 | *Glaucopsyche alexis* | 15,377,669 |
| *Erebia pandrose* | 1,584,267 | *Glaucopsyche melanops* | 1,133,514 |
| *Erebia pharte* | 205,167 | *Gonepteryx cleopatra* | 1,977,950 |
| *Erebia pluto* | 164,522 | *Gonepteryx farinosa* | 2,122,113 |
| *Erebia polaris* | 140,205 | *Gonepteryx rhamni* | 18,516,731 |
| *Erebia pronoe* | 394,213 | *Hamearis lucina* | 4,369,748 |
| *Erebia rhodopensis* | 68,024 | *Hesperia comma* | 35,974,936 |
| *Erebia scipio* | 50,296 | *Heteropterus morpheus* | 9,879,202 |
| *Erebia sthennyo* | 15,948 | *Hipparchia aristaeus* | 33,088 |
| *Erebia stiria* | 74,785 | *Hipparchia blachieri* | 40,809 |
| *Erebia styx* | 107,896 | *Hipparchia cretica* | 8,187 |
| *Erebia sudetica* | 69,897 | *Hipparchia fagi* | 3,413,359 |
| *Erebia triaria* | 285,207 | *Hipparchia fatua* | 2,140,717 |
| *Erebia tyndarus* | 105,117 | *Hipparchia fidia* | 1,042,266 |
| *Erebia zapateri* | 26,910 | *Hipparchia hermione* | 3,342,782 |
| *Erynnis marloyi* | 1,653,261 | *Hipparchia leighebi* | 121 |
| *Erynnis tages* | 8,317,041 | *Hipparchia neomiris* | 33,088 |
| *Euchloe ausonia* | 10,082,254 | *Hipparchia semele* | 5,201,455 |
| *Euchloe bazae* | 32,231 | *Hipparchia senthes* | 615,512 |
| *Euchloe belemia* | 2,920,776 | *Hipparchia statilinus* | 5,153,080 |
| *Euchloe crameri* | 1,650,094 | *Hipparchia syriaca* | 1,775,885 |
| *Euchloe insularis* | 32,877 | *Hipparchia volgensis* | 1,259,729 |
| *Euchloe penia* | 800,716 | *Hyponephele lupina* | 12,022,821 |

**Table S1**. Continued.

| **Species** | **Range size (km²)** | **Species** | **Range size (km²)** |
| --- | --- | --- | --- |
| *Hyponephele lycaon* | 13,875,524 | *Lysandra albicans* | 454,485 |
| *Iolana iolas* | 2,644,191 | *Lysandra bellargus* | 7,019,626 |
| *Iphiclides podalirius* | 10,136,479 | *Lysandra coridon* | 5,084,993 |
| *Issoria lathonia* | 16,163,062 | *Lysandra hispana* | 504,526 |
| *Kirinia climene* | 1,825,148 | *Maniola jurtina* | 11,707,150 |
| *Kirinia roxelana* | 1,295,808 | *Maniola nurag* | 24,097 |
| *Kretania eurypilus* | 1,493,648 | *Melanargia arge* | 103,948 |
| *Kretania hesperica* | 67,420 | *Melanargia galathea* | 6,681,287 |
| *Kretania psylorita* | 8,187 | *Melanargia ines* | 1,230,988 |
| *Kretania sephirus* | 2,718,988 | *Melanargia lachesis* | 652,436 |
| *Kretania trappi* | 29,065 | *Melanargia larissa* | 1,535,500 |
| *Laeosopis roboris* | 681,677 | *Melanargia occitanica* | 1,019,766 |
| *Lasiommata maera* | 12,184,391 | *Melanargia russiae* | 7,796,683 |
| *Lasiommata megera* | 7,824,740 | *Melitaea aetherie* | 515,402 |
| *Lasiommata paramegaera* | 32,877 | *Melitaea arduinna* | 4,171,289 |
| *Lasiommata petropolitana* | 13,043,956 | *Melitaea asteria* | 41,589 |
| *Leptidea duponcheli* | 2,055,402 | *Melitaea athalia* | 19,659,911 |
| *Leptidea morsei* | 12,356,409 | *Melitaea aurelia* | 6,461,032 |
| *Leptidea sinapis* | 15,152,328 | *Melitaea britomartis* | 9,497,360 |
| *Libythea celtis* | 5,042,456 | *Melitaea cinxia* | 14,274,152 |
| *Limenitis camilla* | 8,159,523 | *Melitaea deione* | 897,939 |
| *Limenitis populi* | 19,311,779 | *Melitaea diamina* | 15,231,834 |
| *Limenitis reducta* | 4,657,644 | *Melitaea didyma* | 15,573,420 |
| *Lopinga achine* | 11,523,237 | *Melitaea parthenoides* | 1,117,075 |
| *Lycaena alciphron* | 11,905,690 | *Melitaea phoebe* | 19,974,199 |
| *Lycaena dispar* | 18,088,498 | *Melitaea trivia* | 7,016,376 |
| *Lycaena helle* | 15,110,780 | *Melitaea varia* | 140,775 |
| *Lycaena hippothoe* | 18,252,377 | *Minois dryas* | 14,177,018 |
| *Lycaena ottomana* | 369,062 | *Neolysandra coelestina* | 2,131,703 |
| *Lycaena phlaeas* | 54,357,928 | *Neptis rivularis* | 17,577,567 |
| *Lycaena thersamon* | 9,463,063 | *Neptis sappho* | 13,513,152 |
| *Lycaena thetis* | 641,952 | *Nymphalis c-album* | 28,938,064 |
| *Lycaena tityrus* | 11,505,545 | *Nymphalis egea* | 4,373,937 |
| *Lycaena virgaureae* | 20,151,657 | *Nymphalis polychloros* | 13,522,666 |

**Table S1**. Continued.

| **Species** | **Range size (km²)** | **Species** | **Range size (km²)** |
| --- | --- | --- | --- |
| *Nymphalis vaualbum* | 26,051,813 | *Polyommatus dolus* | 74,391 |
| *Nymphalis xanthomelas* | 24,482,660 | *Polyommatus dorylas* | 5,212,819 |
| *Ochlodes sylvanus* | 17,801,940 | *Polyommatus eros* | 13,702,370 |
| *Oeneis bore* | 12,011,486 | *Polyommatus escheri* | 1,295,373 |
| *Oeneis glacialis* | 135,391 | *Polyommatus fabressei* | 163,715 |
| *Oeneis jutta* | 22,065,659 | *Polyommatus fulgens* | 101,746 |
| *Oeneis norna* | 7,744,405 | *Polyommatus golgus* | 25,848 |
| *Papilio alexanor* | 2,161,613 | *Polyommatus humedasae* | 1,923 |
| *Papilio hospiton* | 32,877 | *Polyommatus icarus* | 29,198,771 |
| *Papilio machaon* | 56,000,322 | *Polyommatus iphigenia* | 740,908 |
| *Pararge aegeria* | 9,841,150 | *Polyommatus nivescens* | 263,314 |
| *Parnassius apollo* | 9,046,555 | *Polyommatus orphicus* | 28,953 |
| *Parnassius mnemosyne* | 7,950,140 | *Polyommatus ripartii* | 6,175,753 |
| *Parnassius phoebus* | 8,094,836 | *Polyommatus thersites* | 10,032,848 |
| *Phengaris alcon* | 12,791,299 | *Polyommatus virgilia* | 65,030 |
| *Phengaris arion* | 15,028,930 | *Pontia callidice* | 18,382,360 |
| *Phengaris nausithous* | 6,842,848 | *Pontia chloridice* | 13,598,717 |
| *Phengaris teleius* | 16,647,079 | *Proterebia afra* | 4,098,084 |
| *Pieris brassicae* | 28,442,059 | *Pseudochazara amymone* | 13,377 |
| *Pieris bryoniae* | 510,114 | *Pseudochazara anthelea* | 1,339,255 |
| *Pieris ergane* | 3,222,439 | *Pseudochazara cingovskii* | 9,356 |
| *Pieris krueperi* | 4,401,486 | *Pseudochazara geyeri* | 579,849 |
| *Pieris mannii* | 4,196,634 | *Pseudochazara graeca* * | 64,544 |
| *Pieris napi* | 43,002,371 | *Pseudochazara orestes* | 8,243 |
| *Pieris rapae* | 38,666,666 | *Pseudochazara tisiphone* | 36,454 |
| *Plebejus argus* | 26,031,761 | *Pseudochazara williamsi* | 21,530 |
| *Plebejus argyrognomon* | 19,688,942 | *Pyrgus alveus* | 14,358,460 |
| *Plebejus idas* | 30,166,657 | *Pyrgus andromedae* | 614,074 |
| *Polyommatus admetus* | 1,290,169 | *Pyrgus armoricanus* | 5,919,449 |
| *Polyommatus amandus* | 18,634,048 | *Pyrgus bellieri* * | 129,695 |
| *Polyommatus aroaniensis* | 88,118 | *Pyrgus cacaliae* | 256,616 |
| *Polyommatus celina* | 1,021,210 | *Pyrgus carlinae* | 162,153 |
| *Polyommatus damon* | 8,185,210 | *Pyrgus carthami* | 8,226,152 |
| *Polyommatus daphnis* | 5,912,898 | *Pyrgus centaureae* | 22,511,006 |

**Table S1**. Continued.

| **Species** | **Range size (km²)** | **Species** | **Range size (km²)** |
| --- | --- | --- | --- |
| *Pyrgus cinarae* | 2,043,203 | *Scolitantides vicrama* | 12,265,710 |
| *Pyrgus cirsii* | 1,703,811 | *Spialia orbifer* | 10,932,149 |
| *Pyrgus malvae* | 20,723,482 | *Spialia phlomidis* | 1,337,724 |
| *Pyrgus onopordi* | 1,285,798 | *Spialia sertorius* | 2,527,291 |
| *Pyrgus serratulae* | 11,358,155 | *Syrichtus cribrellum* | 5,902,932 |
| *Pyrgus sidae* | 7,776,109 | *Syrichtus proto* * | 2,134,296 |
| *Pyrgus warrenensis* | 114,947 | *Syrichtus tessellum* * | 7,704,345 |
| *Pyronia bathseba* | 986,003 | *Tarucus balkanicus* | 10,698,533 |
| *Pyronia cecilia* | 1,949,416 | *Tarucus theophrastus* | 6,854,274 |
| *Pyronia tithonus* | 4,335,208 | *Thecla betulae* | 14,950,976 |
| *Satyrium acaciae* | 5,136,044 | *Thymelicus acteon* | 5,005,843 |
| *Satyrium esculi* | 1,017,798 | *Thymelicus hyrax* | 676,924 |
| *Satyrium ilicis* | 7,154,302 | *Thymelicus lineola* | 22,796,990 |
| *Satyrium pruni* | 14,837,894 | *Thymelicus sylvestris* | 8,540,573 |
| *Satyrium spini* | 7,114,153 | *Tomares ballus* | 930,816 |
| *Satyrium w-album* | 12,263,296 | *Tomares nogelii* | 813,945 |
| *Satyrus actaea* | 569,180 | *Turanana taygetica* | 92,980 |
| *Satyrus ferula* | 6,927,533 | *Zegris eupheme* | 5,273,477 |
| *Scolitantides abencerragus* | 933,869 | *Zerynthia cassandra* | 193,445 |
| *Scolitantides barbagiae* | 3,365 | *Zerynthia cerisyi* | 778,764 |
| *Scolitantides baton* | 1,620,984 | *Zerynthia cretica* | 8,508 |
| *Scolitantides bavius* | 2,478,070 | *Zerynthia polyxena* | 2,911,090 |
| *Scolitantides orion* | 16,638,204 | *Zerynthia rumina* | 1,035,765 |
| *Scolitantides panoptes* | 480,035 | *Zizeeria knysna* | 18,998,999 |

* These five species were initially considered but were discarded from further analyses due to an insufficient number of occupied cells to calculate their climate niche breadths.

**Table S2.** Data sources used for obtaining information on the butterfly’s distributions (range size), larval host plants (diet niche breadth), occupied habitats (habitat niche breadth) and dispersal ability (wingspan).

| **Variable** | **Sources** |
| --- | --- |
| Range size | Fukuda et al. (1994) |
|  | Gorbunov and Kosterin (2004) |
|  | Hesselbarth and van Oorschot (1995) |
|  | Lee (1982) |
|  | Liu (2016) |
|  | Nazari (2003) |
|  | Scott (1986) |
|  | Tennent (1996) |
|  | Tolman (2001) |
|  | Tshikolovets (1998, 2000, 2003, 2005a, 2005b, 2011) |
|  | Tshikolovets, Kosterin, Gorbunov, and Yakovlev (2016) |
|  | Tshikolovets and Pages (2016) |
|  | Tshikolovets, Yakovlev, and Bálint (2009) |
| Diet niche breadth | Bink (1992) |
|  | Bolz and Willig (2014) |
|  | Burnaz (2009) |
|  | Bury (2008) |
|  | Danner (2001) |
|  | Dennis (1992) |
|  | Dumke (2018a, 2018b) |
|  | Ebert (1993a, 1993b) |
|  | Eliasson, Ryrholm, and Gärdenfors (2005) |
|  | Emmet and Heath (1989) |
|  | Fiedler, Konrad (personal communication) |
|  | García-Barros, Munguira, Stefanescu, and Vives Moreno (2014) |
|  | García-Villanueva, Moreno Tamurejo, Vazquez Prado, Nieto Manzano, and Novoa Pérez (2008) |
|  | Garrevoet (1987) |
|  | Gascoigne-Pees, Verovnik, Franeta, and Popović (2014) |
|  | Gascoigne-Pees, Verovnik, Wiskin, Luckens, and Đurić (2012) |
|  | Gorbunov and Kosterin (2004) |
|  | Hensle (2005a, 2005b, 2005c, 2006, 2007a, 2007b, 2007c, 2007d, 2015) |
|  | Hermann (1999) |

**Table S2**. Continued.

| **Variable** | **Sources** |
| --- | --- |
| Diet niche breadth (continued) | Hernández-Roldán, Munguira, Wagner, and Vila (2012) |
|  | Hesselbarth and van Oorschot (1995) |
|  | Huemer (2004) |
|  | Jost (2007) |
|  | Jungklaus (2018) |
|  | Kolev (2017) |
|  | Lafranchis (2016, 2019) |
|  | Lafranchis, Jutzeler, Guillosson, Kan, and Kan (2015) |
|  | Lepidopterologen-Arbeitsgruppe (1987) |
|  | Lepiforum e.V. (2005a, 2005b, 2005c, 2005d) |
|  | Makris (2003) |
|  | Miethke (2017) |
|  | Montiel Pantoja, Martínez Pérez, and Sanjurjo Franch (2020) |
|  | Muñoz Sariot (2013) |
|  | Naderi and Russell (2018) |
|  | Obregón, Fernández Haeger, Marabuto, Fernández, and Jordano (2016) |
|  | Omon (2018) |
|  | Pamperis (1997) |
|  | Paulus (2015) |
|  | Rákosy and Craioveanu (2016) |
|  | Rennwald (2005a, 2005b, 2005c, 2005d, 2005e, 2005f, 2007, 2010, 2017, 2020) |
|  | Robinson, Ackery, Kitching, Beccaloni, and Hernández (2010) |
|  | Savchuk (2005) |
|  | Schweighofer (2005, 2007) |
|  | Schwibinger (2012) |
|  | Scott (1986) |
|  | Settele et al. (2008) |
|  | Sijaric (1976) |
|  | Tarrier and Delacre (2008 ) |
|  | Tennent (1996) |
|  | Tshikolovets (2011) |
|  | UK Butterflies (2020) |

**Table S2**. Continued.

| **Variable** | **Sources** |
| --- | --- |
| Diet niche breadth (continued) | Villa (1999) |
|  | Wagner (2009, 2020a, 2020b, 2020c, 2020d, 2020e, 2020f, 2020g, 2020h) |
|  | Welz (2014) |
|  | Woodhall (2005) |
|  | Zhakov (2011) |
|  | Ziegler (2010, 2019a, 2019b) |
| Habitat niche breadth | Emmet and Heath (1989) |
|  | Settele, Feldmann, and Reinhardt (1999) |
|  | Tshikolovets (2011) |
| Wingspan | Bink (1992) |
|  | Emmet and Heath (1989) |
|  | García-Barros, Munguira, Stefanescu, and Vives Moreno (2014) |
|  | Fiedler, Konrad (personal communication) |
|  | Makris (2003) |
|  | Pamperis (1997) |
|  | Tshikolovets (2011) |

**Table S2 References:**

Bink, F. A. (1992). *Ecologische Atlas van de Dagvlinders van Noordwest-Europa*. Haarlem, NL: Schuyt & Co.

Bolz, R., & Willig, S. (2014). Ergänzungen zum Atlas „Tagfalter in Bayern“: Kontinentale Steppenpflanzen als Nahrungspflanzen für heimische Tagfalter (Lepidoptera: Lycaenidae). *Nachrichten des entomologischen Vereins Apollo, 35*, 149–151.

Burnaz, S. (2009). Macrolepidoptera species characteristic for the montane, subalpine and alpine levels of the massifs situated in Hunedoara County (Romania). *Muzeul Olteniei Craiova. Oltenia. Studii şi comunicari. Ştiințele Naturii, 25*, 145–152.

Bury, J. (2008). *Maculinea arion* (LINNAEUS, 1758) *Foto*. Retrieved from https://www.lepiforum.de/2_forum.pl?md=read;id=31176

Danner, F. (2001). Die Raupe von *Charaxes jasius* (Linnaeus, 1767) auf Aprikose (Lepidoptera, Nymphalidae). *Atalanta, 32*, 401.

Dennis, R. L. H. (Ed.) (1992). *The ecology of butterflies in Britain*. Oxford, UK: Oxford University Press.

Dumke, M. (2018a). *Euphydryas cynthia* *Foto*. Retrieved from https://www.lepiforum.de/2_forum_2017.pl?page=1;md=read;id=1012

Dumke, M. (2018b). *Melitaea phoebe* *Foto*. Retrieved from https://www.lepiforum.de/2_forum_2017.pl?page=1;md=read;id=10135

Ebert, G. (Ed.) (1993a). *Die Schmetterlinge Baden-Württembergs, Band 1 Tagfalter I*. Stuttgart, DE: Verlag Eugen Ulmer.

Ebert, G. (Ed.) (1993b). *Die Schmetterlinge Baden-Württembergs, Band 2 Tagfalter II*. Stuttgart, DE: Verlag Eugen Ulmer.

Eliasson, C. U., Ryrholm, N., & Gärdenfors, U. (2005). *Nationalnyckeln till Sveriges flora och fauna: Fjärilar: Dagfjärilar: Hesperiidae-Nymphalidae*. Uppsala, SE: ArtDatabanken SLU.

Emmet, A. M., & Heath, J. (Eds.). (1989). *The butterflies of Great Britain and Ireland, Volume 7, Part 1: Hesperiidae - Nymphalidae*. Colchester, UK: Harley Books.

Fukuda, H., Hama, E., Kuzuya, T., Takahashi, A., Takahashi, M., Tanaka, H., . . . Watanabe, Y. (1994). *The life histories of butterflies in Japan* (4th ed. Vol. 1–4). Osaka, JP: Hoikusha Publishing Co.

García-Barros, E., Munguira, M., Stefanescu, C., & Vives Moreno, A. (2014). *Fauna Ibérica, Volume 37: Lepidoptera: Papilionoidea*. Madrid, ES: Consejo Superior de Investigaciones Científicas.

García-Villanueva, V., Moreno Tamurejo, J. A., Vazquez Prado, F. M., Nieto Manzano, M. A., & Novoa Pérez, J. M. (2008). *Melitaea aetherie* (Hübner, 1826) en la provincia de Badajoz (España): nuevos datos sobre su biología y distribución (Lepidoptera: Nymphalidae). *Boletín Sociedad Entomológica Aragonesa, 42*, 279–288.

Garrevoet, T. (1987). The breeding of *Pseudochazara orestes* De Prins & van der Poorten, 1981 and description of the pre-imaginal stadia (Lepidoptera : Nymphalidae : Satyrinae). *Phegea, 15*, 1–7.

Gascoigne-Pees, M., Verovnik, R., Franeta, F., & Popović, M. (2014). The lifecycle and ecology of *Pseudochazara amymone* (Brown, 1976) (Lepidoptera: Nymphalidae, Satyrinae). *Nachrichten des entomologischen Vereins Apollo, 35*, 129–138.

Gascoigne-Pees, M., Verovnik, R., Wiskin, C., Luckens, C., & Đurić, M. (2012). Notes on the lifecycle of *Melitaea arduinna* (Esper, 1783) (“Freyer’s fritillary”) (Lepidoptera: Nymphalidae) with further records from SE Serbia. *Nachrichten des entomologischen Vereins Apollo, 33*, 9–14.

Gorbunov, P. I., & Kosterin, O. E. (2004). *The Butterflies of North Asia (Asian part of Russia) in Nature.* (Vol. 1–2). Moscow, RU: Rodina & Fodio.

Hensle, J. (2005a). *Boloria pales* (Denis & Schiffermüller, 1775), Lebensweise. Retrieved from https://lepiforum.org/wiki/page/Boloria_pales

Hensle, J. (2005b). *Issoria lathonia* (Linnaeus, 1758), Nahrung der Raupe. Retrieved from https://lepiforum.org/wiki/page/Issoria_lathonia

Hensle, J. (2005c). *Pieris bryoniae* (Hübner, 1806), Biologie. Retrieved from https://lepiforum.org/wiki/page/Pieris_bryoniae

Hensle, J. (2006). *Vanessa virginiensis* (Drury, 1773), Lebensraum und Lebensweise. Retrieved from https://lepiforum.org/wiki/page/Vanessa_virginiensis

Hensle, J. (2007a). *Archon apollinus* (Herbst, 1798), Nahrung der Raupe. Retrieved from https://lepiforum.org/wiki/page/Archon_apollinus

Hensle, J. (2007b). *Euchloe insularis* (Staudinger, 1861), Lebensweise. Retrieved from https://lepiforum.org/wiki/page/Euchloe_insularis

Hensle, J. (2007c). *Euphydryas desfontainii* (Godart, 1819), Lebensweise. Retrieved from https://lepiforum.org/wiki/page/Euphydryas_desfontainii

Hensle, J. (2007d). *Gonepteryx farinosa* (Zeller, 1847), Lebensweise. Retrieved from https://lepiforum.org/wiki/page/Gonepteryx_farinosa

Hensle, J. (2015). *Erebia sudetica* *Foto*. Retrieved from https://www.lepiforum.de/2_forum_2013.pl?page=1;md=read;id=21499

Hermann, G. (1999). Neue Beobachtungen zu Eiablage- und Raupennahrungspflanzen von Tagfalterarten in Baden-Württemberg (Lepidoptera, Rhopalocera). *Atalanta, 29*, 245–254.

Hernández-Roldán, J. L., Munguira, M. L., Wagner, W., & Vila, R. (2012). Comparative analysis and taxonomic use of the morphology of immature stages and natural history traits in European species of *Pyrgus* Hübner (Lepidoptera: Hesperiidae, Pyrginae). *Zootaxa, 3470*, 1–71.

Hesselbarth, G., & van Oorschot, H. W., S. (1995). *Die Tagfalter der Türkei unter Berücksichtigung der angrenzenden Länder* (Vol. 1–3). Bocholt, DE: Selbstverlag Sigbert Wagener.

Huemer, P. (2004). *Die Tagfalter Südtirols*. Vienna, AT: Folio Verlag.

Jost, B. (2007). *Melitaea deione* Futterpflanze BH *Bild*. Retrieved from https://www.lepiforum.de/2_forum.pl?md=read;id=10312

Jungklaus, O. (2018). *Melitaea*-Raupen? *Foto*. Retrieved from https://www.lepiforum.de/1_forum_2018.pl?page=1;md=read;id=22006

Kolev, Z. (2017). *Rubrapterus bavius* (Eversmann, 1832), a butterfly genus and species new to Bulgaria (Insecta, Lepidoptera, Lycaenidae). *ZooNotes, 114*, 1–4. https://doi.org/10.24193/entomolrom.21.3

Lafranchis, T. (2016). Raupen und Puppe von *Polyommatus aroaniensis* *Foto*. Retrieved from https://www.lepiforum.de/2_forum_2013.pl?page=1;md=read;id=24469

Lafranchis, T. (2019). Notes on the biology of some butterflies in Greece (Lepidoptera: Papilionoidea). *Entomologist's Gazette, 70*, 113–134. https://doi.org/10.31184/G00138894.702.1710

Lafranchis, T., Jutzeler, D., Guillosson, J.-Y., Kan, P., & Kan, B. (2015). *La vie des papillons: Ecologie, biologie et comportement des rhopalocères de France*. Paris, FR: Diatheo.

Lee, S. M. (1982). *Butterflies of Korea*. Seoul, KR: Editorial Committee of Insecta Koreana.

Lepidopterologen-Arbeitsgruppe. (1987). *Tagfalter und ihre Lebensräume: Arten, Gefährdung, Schutz. Band 1*. Basel, CH: Schweizerischer Bund für Naturschutz.

Lepiforum e.V. (2005a). *Melitaea asteria* (Freyer, 1828), Lebensraum und Lebensweise. Retrieved from https://lepiforum.org/wiki/page/Melitaea_asteria

Lepiforum e.V. (2005b). *Melitaea britomartis* (Assmann, 1847). Retrieved from https://lepiforum.org/wiki/page/Melitaea_britomartis

Lepiforum e.V. (2005c). *Melitaea deione* (Geyer, 1832), Lebensweise. Retrieved from https://lepiforum.org/wiki/page/Melitaea_deione

Lepiforum e.V. (2005d). *Pyrgus onopordi* (Rambur, 1839), Nahrung der Raupe. Retrieved from https://lepiforum.org/wiki/page/Pyrgus_onopordi

Liu, Z.-H. (2016). A contribution to the butterfly fauna of S. Gansu, China. *Atalanta, 47*, 249–254.

Makris, C. (2003). *Butterflies of Cyprus*. Nicosia, CY: Bank of Cyprus Cultural Foundation.

Miethke, M. (2017). *Phengaris arion* (Linnaeus, 1758), Eiablage. Retrieved from https://lepiforum.org/wiki/page/Phengaris_arion

Montiel Pantoja, C., Martínez Pérez, I., & Sanjurjo Franch, M. J. (2020). Notas sobre la biología, ecología y distribución de *Pieris ergane* (Geyer, 1828) (Lepidoptera: Pieridae) en la Cordillera Cantábrica, provincia de León (noroeste de España). *Arquivos Entomolóxicos, 22*, 423–444.

Muñoz Sariot, M. G. (2013). Ciclo biológico, morfología de los estadios preimaginales y nuevos datos sobre la distribución de *Borbo borbonica zelleri* (Lederer, 1855) (Lepidoptera:Hesperiidae) en la provincia de Cádiz, España. *Revista gaditana de Entomología, 4*, 137–158.

Naderi, A. R., & Russell, P. (2018). The larva and a new host-plant of *Melitaea arduinna* (Lepidoptera: Nymphalidae) in Iran. *Phegea, 46*, 137–138.

Nazari, V. (2003). *Butterflies of Iran*. Tehran, IR: Department of Environment, Dayere Sabz Publications.

Obregón, R., Fernández Haeger, J., Marabuto, E., Fernández, P., & Jordano, D. (2016). *Distribución potencial y efecto del cambio climático sobre el ninfálido Melitaea aetherie (Lepidoptera, Nymphalidae) en el Mediterráneo occidental*. Paper presented at the IX Congreso Español de Biogeografía, Algeciras, ES.

Omon, B. (2018). *Melitaea phoebe* *Foto*. Retrieved from https://www.lepiforum.de/2_forum_2017.pl?page=1;md=read;id=11728

Pamperis, L. N. (1997). *The butterflies of Greece* (1st ed.). Athens, GR: Bastas-Plessas.

Paulus, G. (2015). Re: Fotonachtrag *E.* [a.] *beckeri* - Raupe *Foto*. Retrieved from https://www.lepiforum.de/2_forum_2013.pl?page=1;md=read;id=19361

Rákosy, L., & Craioveanu, C. (2016). Redescovering *Tomares nogelii dobrogensis* Caradja, 1895 in Romania. *Entomologica Romanica, 19*, 13–16.

Rennwald, E. (2005a). *Carcharodus alceae* (Esper, 1780), Nahrung der Raupe. Retrieved from https://lepiforum.org/wiki/page/Carcharodus_alceae

Rennwald, E. (2005b). *Colias alfacariensis* (Ribbe, 1905), Nahrung der Raupe. Retrieved from https://lepiforum.org/wiki/page/Colias_alfacariensis

Rennwald, E. (2005c). *Pyrgus armoricanus* (Oberthür, 1910), Nahrung der Raupe. Retrieved from https://lepiforum.org/wiki/page/Pyrgus_armoricanus

Rennwald, E. (2005d). *Pyrgus carlinae* (Rambur, 1839), Nahrung der Raupe. Retrieved from https://lepiforum.org/wiki/page/Pyrgus_carlinae

Rennwald, E. (2005e). *Pyrgus cirsii* (Rambur, 1839), Nahrung der Raupe. Retrieved from https://lepiforum.org/wiki/page/Pyrgus_cirsii

Rennwald, E. (2005f). *Pyrgus serratulae* (Rambur, 1839), Nahrung der Raupe. Retrieved from https://lepiforum.org/wiki/page/Pyrgus_serratulae

Rennwald, E. (2007). *Erynnis marloyi* (Boisduval, 1834), Nahrung der Raupe. Retrieved from https://lepiforum.org/wiki/page/Erynnis_marloyi

Rennwald, E. (2010). *Pyrgus centaureae* (Rambur, 1839), Nahrung der Raupe. Retrieved from https://lepiforum.org/wiki/page/Pyrgus_centaureae

Rennwald, E. (2017). *Phengaris alcon* (Denis & Schiffermüller, 1775), Taxonomie und Nomenklatur. Retrieved from https://lepiforum.org/wiki/page/Phengaris_alcon

Rennwald, E. (2020). *Muschampia orientalis* (Reverdin, 1913), Nahrung der Raupe. Retrieved from https://lepiforum.org/wiki/page/Muschampia_orientalis

Robinson, G. S., Ackery, P. R., Kitching, I. J., Beccaloni, G. W., & Hernández, L. M. (2010). HOSTS - a database of the world's lepidopteran hostplants. Retrieved from http://www.nhm.ac.uk/hosts

Savchuk, V. (2005). *Chazara Persephone*. Retrieved from https://lepidoptera.crimea.ua/2705_Satyridae/Chazara_persephone.htm

Schweighofer, W. (2005). *Melitaea phoebe* (Denis & Schiffermüller, 1775), Eiablage. Retrieved from https://lepiforum.org/wiki/page/Melitaea_phoebe

Schweighofer, W. (2007). *Pyrgus armoricanus*, 3. Generation *Bild*. Retrieved from https://www.lepiforum.de/2_forum.pl?md=read;id=16532

Schwibinger, M. (2012). Re: *Euphydryas cynthia* - Raupen fressend *Bild* *Foto*. Retrieved from https://www.lepiforum.de/2_forum.pl?md=read;id=84198

Scott, J. A. (1986). *The butterflies of North America: a natural history and field guide*. Stanford, CA: Stanford University Press.

Settele, J., Feldmann, R., & Reinhardt, R. (1999). *Die Tagfalter Deutschlands*. Stuttgart, DE: Verlag Eugen Ulmer.

Settele, J., Kudrna, O., Harpke, A., Kühn, I., Van Swaay, C., Verovnik, R., . . . Schweiger, O. (2008). *Climatic risk atlas of European butterflies*. Sofia, BG: Pensoft Publishers.

Sijaric, R. (1976). Interspezifische Verhältnisse der Arten *Coenonympha tullia* Müller und *C. rhodopensi*s Elwes auf der Balkanhalbinsel. *Wissenschaftliche Mitteilungen des Bosnisch-Herzegowinischen Landesmuseums, 6*, 133–177.

Tarrier, M. R., & Delacre, J. (2008 ). *Les Papillons de jour du Maroc: Guide d'identification et de bio-indication*. Paris, FR: Biotope, Publications scientifiques du MNHN.

Tennent, J. W. (1996). *The butterflies of Morocco, Algeria and Tunisia*. Wallingford, Oxfordshire, UK: Gem Publishing Company.

Tolman, T. (2001). *Butterflies of Europe*. Princeton, NJ: Princeton University Press.

Tshikolovets, V. V. (1998). *The butterflies of Turkmenistan*. Kyiv, Brno: Tshikolovets Publications.

Tshikolovets, V. V. (2000). *The butterflies of Uzbekistan*. Kyiv, Brno: Tshikolovets Publications.

Tshikolovets, V. V. (2003). *The butterflies of Tajikistan*. Kyiv, Brno: Tshikolovets Publications.

Tshikolovets, V. V. (2005a). *The butterflies of Kyrgyzstan*. Kyiv, Brno: Tshikolovets Publications.

Tshikolovets, V. V. (2005b). *The butterflies of Ladak (N.-W. India)*. Kyiv, Brno: Tshikolovets Publications.

Tshikolovets, V. V. (2011). *Butterflies of Europe & the Mediterranean area*. Pardubice, CZ: Tshikolovets Publications.

Tshikolovets, V. V., Kosterin, O. E., Gorbunov, P. I., & Yakovlev, R. V. (2016). *The butterflies of Kazakhstan*. Pardubice, CZ: Tshikolovets Publications.

Tshikolovets, V. V., & Pages, J. (2016). *The butterflies of Pakistan* (Vol. 12). Paradubice, CZ: Tshikolovets Publications.

Tshikolovets, V. V., Yakovlev, R. V., & Bálint, Z. (2009). *The butterflies of Mongolia*. Pardubice, CZ: Tshikolovets Publications.

UK Butterflies. (2020). Grizzled skipper - *Pyrgus malvae*. Retrieved from https://www.ukbutterflies.co.uk/species.php?species=malvae

Villa, R. (1999). Influence of day-length on seasonal dimorphism of *Polygonia egea* (Cramer, 1775) (Insecta Lepidoptera Nymphalidae). *Quaderno di Studi e Notizie di Storia Naturale della Romagna, 11*, 63–69.

Wagner, W. (2009). Zur Freiland-Raupennahrung von *Pyrgus onopordi* (Rambur 1839) in Andalusien (Lepidoptera: Hesperiidae). *Nachrichten des entomologischen Vereins Apollo, 30*, 1–4.

Wagner, W. (2020a). *Carcharodus baeticus* (Rambur, 1839) (Andorn-Dickkopf). Retrieved from http://www.pyrgus.de/Carcharodus_baeticus.html

Wagner, W. (2020b). *Laeosopis roboris* (Esper, 1793) (Eschen-Zipfelfalter). Retrieved from http://pyrgus.de/Laeosopis_roboris.html

Wagner, W. (2020c). *Leptidea duponcheli* (Staudinger, 1871). Retrieved from http://pyrgus.de/Leptidea_duponcheli.html

Wagner, W. (2020d). *Pieris krueperi* (Staudinger, 1860). Retrieved from http://pyrgus.de/Pieris_krueperi.html

Wagner, W. (2020e). *Polyommatus glandon* (Prunner, 1798) (Mannsschild-Bläuling). Retrieved from http://pyrgus.de/Polyommatus_glandon.html

Wagner, W. (2020f). *Polyommatus nicias* (Meigen, 1830). Retrieved from http://pyrgus.de/Polyommatus_nicias.html

Wagner, W. (2020g). *Pyrgus carthami* (Hübner, 1813) (Steppenheiden-Würfeldickkopf). Retrieved from http://pyrgus.de/Pyrgus_carthami.html

Wagner, W. (2020h). *Spialia orbifer* (Hübner, 1823). Retrieved from http://pyrgus.de/Spialia_orbifer.html

Welz, M. (2014). Stachelige Raupe mit gelben Querstreifen *Foto*. Retrieved from https://www.lepiforum.de/1_forum.pl?page=1;md=read;id=89622

Woodhall, S. (2005). *Field guide to butterflies of South Africa*. Cape Town, ZA: Struik.

Zhakov, A. (2011). *Tomares nogelii* Eiablage an Astragalus ponticus *Bild* *Foto*. Retrieved from https://www.lepiforum.de/2_forum.pl?md=read;id=81955

Ziegler, H. (2010). *Euchloe ausonia taurica*: L5 *Bild*. Retrieved from https://www.lepiforum.de/2_forum.pl?md=read;id=58942

Ziegler, H. (2019a). *Leptidea duponcheli* (STAUDINGER, 1871). Retrieved from http://euroleps.ch/seiten/s_art.php?art=pier_duponcheli

Ziegler, H. (2019b). *Pseudophilotes vicrama schiffermuelleri* (HEMMING, 1929). Retrieved from http://euroleps.ch/seiten/s_art.php?art=lyc_schiffermuelleri

**Table S3.** Principal component analysis (PCA) based on 19 bioclimatic variables downloaded from the CHELSA database (Karger et al., 2017a, 2017b). Values represent the loadings of the first four principal components used for calculating the species’ climate niche breadth.

| **Variable** | | **Principal component** | | | |
| --- | --- | --- | --- | --- | --- |
|  |  | **PC1** | **PC2** | **PC3** | **PC4** |
| BIO1 | Annual Mean Temperature | 0.28 | 0.19 | 0.05 | 0.07 |
| BIO2 | Mean Diurnal Range | 0.13 | 0.41 | 0.08 | 0.00 |
| BIO3 | Isothermality | 0.27 | 0.04 | −0.27 | 0.01 |
| BIO4 | Temperature Seasonality | −0.22 | 0.10 | 0.48 | −0.12 |
| BIO5 | Max. Temperature of Warmest Month | 0.25 | 0.27 | 0.19 | 0.04 |
| BIO6 | Min. Temperature of Coldest Month | 0.30 | 0.12 | −0.09 | 0.09 |
| BIO7 | Temperature Annual Range | −0.17 | 0.22 | 0.51 | −0.13 |
| BIO8 | Mean Temperature of Wettest Quarter | 0.25 | 0.22 | 0.24 | −0.05 |
| BIO9 | Mean Temperature of Driest Quarter | 0.27 | 0.14 | −0.14 | 0.15 |
| BIO10 | Mean Temperature of Warmest Quarter | 0.26 | 0.25 | 0.18 | 0.04 |
| BIO11 | Mean Temperature of Coldest Quarter | 0.29 | 0.14 | −0.08 | 0.08 |
| BIO12 | Annual Precipitation | 0.25 | −0.28 | 0.09 | −0.17 |
| BIO13 | Precipitation of Wettest Month | 0.24 | −0.19 | 0.02 | −0.39 |
| BIO14 | Precipitation of Driest Month | 0.16 | −0.33 | 0.27 | 0.25 |
| BIO15 | Precipitation Seasonality | −0.03 | 0.16 | −0.21 | −0.62 |
| BIO16 | Precipitation of Wettest Quarter | 0.24 | −0.19 | 0.02 | −0.38 |
| BIO17 | Precipitation of Driest Quarter | 0.17 | −0.33 | 0.26 | 0.24 |
| BIO18 | Precipitation of Warmest Quarter | 0.19 | −0.20 | 0.29 | −0.28 |
| BIO19 | Precipitation of Coldest Quarter | 0.18 | −0.27 | 0.00 | 0.06 |

**Table S4.** List of BOLD ProcessIDs and Genbank accession numbers for all sequences used to reconstruct the butterfly phylogeny. Note that some species were later pruned from the tree or merged with other species (see footnotes). COI, cytochrome c oxidase subunit I; WGL, wingless; EF1A, elongation factor 1-alpha; RPS5, ribosomal protein S5; GAPDH, glyceraldehyde 3-phosphate dehydrogenase; CAD, carbamoyl-phosphate synthetase 2; IDH, isocitrate dehydrogenase.

| **Species** | **COI** | **WGL** | **EF1A** | **RPS5** | **GAPDH** | **CAD** | **IDH** |
| --- | --- | --- | --- | --- | --- | --- | --- |
| *Aglais io* | LOWA167-06 | AF412766.1 | AY248810.1 | FJ639576.1 | FJ639521.1 |  |  |
| *Aglais urticae* | GBGL31771-19 | KJ649130.1 | AY248811.1 | FJ639577.1 | FJ639522.1 | GQ864599.1 | GQ865055.1 |
| *Agriades aquilo* | RDHP383-06 |  |  |  |  |  |  |
| *Agriades glandon* | GBGL6590-09 |  | EU326285.1 |  |  |  |  |
| *Agriades optilete* | LYCAE090-15 | JX093444.1 | GQ128699.1 |  |  | GQ128630.1 |  |
| *Agriades orbitulus* | EZHBA673-07 | GQ128842.1 | GQ128634.1 |  |  | GQ128560.1 |  |
| *Agriades pyrenaica* | GBGLL197-13 |  |  |  |  |  |  |
| *Agriades zullichi* | EZSPN659-09 |  |  |  |  |  |  |
| *Anthocharis cardamines* | LOWA322-06 | KM046563.1 | KM669649.1 | KM046601.1 | KM046841.1 | KM046912.1 | KM046735.1 |
| *Anthocharis damone* | GBGL4364-07 |  |  |  |  |  |  |
| *Anthocharis euphenoides* | EZSPN090-09 |  |  |  |  |  |  |
| *Anthocharis gruneri* | LOWA560-06 |  |  |  |  |  |  |
| *Apatura ilia* | GBLN1813-08 |  | JX185969.1 |  |  |  |  |
| *Apatura iris* | GBLN1814-08 | AY090132.1 | AY090165.1 | EU141413.1 | EU141513.1 | EU141335.1 | EU141572.1 |
| *Apatura metis* | EZROM701-08 |  | GU372611.1 |  |  |  |  |
| *Aphantopus hyperantus* | GBLN0144-06 | AY090144.1 | AY090177.2 | GQ357588.1 | GQ357458.1 | JN204940.1 | JN205006.1 |
| *Aporia crataegi* | GBGL4974-08 | DQ082806.1 | AB069900.1 | EU141394.1 | EU141496.1 | EU141316.1 | EU141553.1 |
| *Araschnia levana* | GBLN2880-10 | AF412762.1 | AY248805.1 | GQ865391.1 | GQ864925.1 | GQ864610.1 | GQ865064.1 |
| *Archon apollinus* | GBGL4842-08 | DQ351135.1 | DQ351111.1 |  |  |  |  |

**Table S4**. Continued.

| **Species** | **COI** | **WGL** | **EF1A** | **RPS5** | **GAPDH** | **CAD** | **IDH** |
| --- | --- | --- | --- | --- | --- | --- | --- |
| *Arethusana arethusa* | GBLN0780-06 | DQ338728.1 | DQ339018.1 | GQ357626.1 | GQ357500.1 |  |  |
| *Argynnis adippe* | GBLN0824-06 | DQ922820.1 | DQ922884.1 | KY773505.1 | KY773401.1 |  |  |
| *Argynnis aglaja* | GBLN0832-06 | DQ922828.1 | DQ922892.1 | KY773492.1 | KY773387.1 |  |  |
| *Argynnis elisa* | GBGL29402-19 | KY773445.1 | KY773341.1 | KY773498.1 | KY773393.1 |  |  |
| *Argynnis laodice* | GBLN0816-06 | DQ922812.1 | GU372619.1 | KY773485.1 | KY773381.1 |  |  |
| *Argynnis niobe* | GBLN0823-06 | DQ922819.1 | DQ922883.1 |  | KY773439.1 |  |  |
| *Argynnis pandora* | GBLN0834-06 | DQ922830.1 | DQ922894.1 | KY773486.1 | KY773382.1 |  |  |
| *Argynnis paphia* | GBLN0133-06 | AF412778.1 | AY090166.1 | EU141421.1 | EU141519.1 | EU141343.1 | EU141580.1 |
| *Aricia agestis* | EZROM039-08 | GQ128843.1 | AY496824.1 |  |  | GQ128561.1 |  |
| *Aricia anteros* | GBGL0894-06 |  | KJ671880.1 |  |  |  |  |
| *Aricia artaxerxes* | GBGL0854-06 | JX093415.1 | JX093308.1 |  |  | JX093255.1 |  |
| *Aricia cramera* | GBGL0833-06 |  |  |  |  |  |  |
| *Aricia eumedon* | GBGL0930-06 | GQ128851.1 | GQ128642.1 |  |  | GQ128570.1 |  |
| *Aricia montensis* | EZSPC191-09 |  |  |  |  |  |  |
| *Aricia morronensis* | EZSPN076-09 |  |  |  |  |  |  |
| *Aricia nicias* | EZSPM551-10 | GQ128892.1 | GQ128681.1 |  |  | GQ128612.1 |  |
| *Boloria aquilonaris* | GBLN0839-06 | DQ922835.1 | DQ922899.1 |  |  |  |  |
| *Boloria chariclea* | GBMIN37591-13 | HQ161168.1 | HQ161295.1 |  |  |  |  |
| *Boloria dia* | LOWA090-06 | HQ161216.1 | HQ161353.1 |  |  |  |  |
| *Boloria eunomia* | GBLN0841-06 | DQ922837.1 | DQ922901.1 |  |  |  |  |
| *Boloria euphrosyne* | GBLN0840-06 | DQ922836.1 | DQ922900.1 |  |  |  |  |

**Table S4**. Continued.

| **Species** | **COI** | **WGL** | **EF1A** | **RPS5** | **GAPDH** | **CAD** | **IDH** |
| --- | --- | --- | --- | --- | --- | --- | --- |
| *Boloria freija* | EZHBA856-07 | HQ161169.1 | HQ161296.1 |  |  |  |  |
| *Boloria frigga* | GUPPY606-18 | HQ161170.1 | HQ161297.1 |  |  |  |  |
| *Boloria graeca* | PHLAF404-11 | HQ161193.1 | HQ161324.1 |  |  |  |  |
| *Boloria improba* | HBNK493-07 | HQ161166.1 | HQ161293.1 |  |  |  |  |
| *Boloria napaea* | GBLN2869-10 | GQ864434.1 | GQ864840.1 | GQ865404.1 |  | GQ864621.1 | GQ865074.1 |
| *Boloria pales* | GBLN0838-06 | DQ922834.1 | DQ922898.1 |  |  |  |  |
| *Boloria polaris* | HBNK255-07 | HQ266646.1 | HQ266658.1 |  |  |  |  |
| *Boloria selene* | GBLN0134-06 | HQ161167.1 | HQ161294.1 |  |  |  |  |
| *Boloria thore* | HBNK076-07 | HQ161207.1 | HQ161344.1 |  |  |  |  |
| *Boloria titania* | LOWA219-06 | HQ161188.1 | HQ161317.1 |  |  |  |  |
| *Borbo borbonica* | EULEP226-14 |  |  |  |  |  |  |
| *Brenthis daphne* | GBLN0820-06 | DQ922816.1 | DQ922880.1 | KY773533.1 | KY773429.1 | MH028651.1 |  |
| *Brenthis hecate* | GBLN0818-06 | DQ922814.1 | DQ922878.1 | KY773491.1 | KY773386.1 | MH028674.1 |  |
| *Brenthis ino* | GBLN0819-06 | DQ922815.1 | DQ922879.1 | GQ865405.1 | KY773400.1 | GQ864622.1 |  |
| *Brintesia circe* | GBLN0782-06 | DQ338729.1 | DQ339020.1 | EU141370.1 | EU141474.1 | EU141291.1 | EU141527.1 |
| *Callophrys avis* | EZSPN615-09 |  |  |  |  |  |  |
| *Callophrys rubi* | EZROM071-08 |  |  |  |  |  |  |
| *Carcharodus alceae* | EZROM073-08 | EU363982.1 | EU364175.1 | KY028527.1 |  | KY045551.1 | KY027761.1 |
| *Carcharodus baeticus* | EZSPN141-09 |  |  |  |  |  |  |
| *Carcharodus flocciferus* | EZROM613-08 |  |  |  |  |  |  |
| *Carcharodus lavatherae* | LOWAB072-07 |  |  |  |  |  |  |

**Table S4**. Continued.

| **Species** | **COI** | **WGL** | **EF1A** | **RPS5** | **GAPDH** | **CAD** | **IDH** |
| --- | --- | --- | --- | --- | --- | --- | --- |
| *Carcharodus orientalis* | GBGL36352-19 |  |  |  |  |  |  |
| *Carterocephalus palaemon* | GBGL30293-19 | EU363990.1 | EU364183.1 | KY028529.1 | KY027506.1 | KY045552.1 | KY027763.1 |
| *Carterocephalus silvicolus* | GBGL0054-06 | JN204921.1 | GU372683.1 | JN205030.1 |  | JN204936.1 | JN205003.1 |
| *Celastrina argiolus* | GBGL34983-19 |  | KJ671882.1 |  |  |  |  |
| *Charaxes jasius* | EZCHA046-07 | GQ256703.1 | GQ256948.1 | GQ257150.1 |  |  |  |
| *Chazara briseis* | GBLN0783-06 | DQ338730.1 | DQ339021.1 | GQ357628.1 | GQ357502.1 |  |  |
| *Chazara persephone* | LOWAB005-07 |  |  |  |  |  |  |
| *Chazara prieuri* | EZSPC805-10 |  |  |  |  |  |  |
| *Chilades trochylus* | GBGL34985-19 | GQ128853.1 | GQ128644.1 |  |  | GQ128572.1 |  |
| *Coenonympha arcania* | LOWAB061-07 | EU920807.1 | EU920777.1 |  |  |  |  |
| *Coenonympha corinna* | OXB894-15 | EU920809.1 | EU920779.1 |  |  |  |  |
| *Coenonympha darwiniana* ^1^ | LEPAA106-16 |  |  |  |  |  |  |
| *Coenonympha dorus* | EZSPN201-09 | EU920810.1 | EU920780.1 |  |  |  |  |
| *Coenonympha gardetta* ^1^ | PHLAA709-09 | EU920811.1 | EU920781.1 |  |  |  |  |
| *Coenonympha glycerion* | GBLN0312-06 | EU920812.1 | EU920783.1 | GQ892095.1 | GQ892089.1 |  |  |
| *Coenonympha hero* | GBLN0310-06 | DQ338636.1 | DQ338919.1 |  |  |  |  |
| *Coenonympha leander* | IRANB238-08 |  |  |  |  |  |  |
| *Coenonympha oedippus* | PHLSA390-11 | EU920817.1 | EU920788.1 | GQ892096.1 |  |  |  |
| *Coenonympha pamphilus* | GBLN0695-06 | DQ338637.1 | DQ338920.1 | EU528428.1 | EU528385.1 |  |  |
| *Coenonympha rhodopensis* | EZROM880-08 | EU920818.1 | EU920789.1 | MH157717.1 | MH157708.1 |  |  |
| *Coenonympha tullia* | GBLN2931-10 | DQ351126.1 | AF173399.2 | KF721248.1 |  |  |  |

**Table S4**. Continued.

| **Species** | **COI** | **WGL** | **EF1A** | **RPS5** | **GAPDH** | **CAD** | **IDH** |
| --- | --- | --- | --- | --- | --- | --- | --- |
| *Colias alfacariensis* | FBLMZ482-12 |  |  |  |  |  |  |
| *Colias aurorina* | IRANB306-08 |  | EF457742.1 |  |  |  |  |
| *Colias caucasica* | AF073943.1 |  |  |  |  |  |  |
| *Colias chrysotheme* | EZROM119-08 |  |  |  |  |  |  |
| *Colias crocea* * | LOWA288-06 |  | EF457747.1 |  |  | MF185821.1 |  |
| *Colias erate* | GBGL4829-08 | HM236308.1 | EF457745.1 |  |  | MF185825.1 |  |
| *Colias hecla* | EZHBA860-07 |  |  |  |  |  |  |
| *Colias hyale* | GBGL4826-08 |  | EF457744.1 |  |  |  |  |
| *Colias myrmidone* | EZROM131-08 |  |  |  |  |  |  |
| *Colias palaeno* | LCH001-04 | GU829570.1 | GU829301.1 | GU830680.1 | GU829810.1 | GU828181.1 | GU830077.1 |
| *Colias phicomone* | PHLAA141-09 |  |  |  |  |  |  |
| *Colias tyche* | LOWA744-06 |  |  |  |  |  |  |
| *Cupido alcetas* | EZSPC407-09 | KP824791.1 | KJ671883.1 |  |  |  |  |
| *Cupido argiades* | EZROM582-08 |  | GU372629.1 |  |  |  |  |
| *Cupido carswelli* | EZSPC1405-10 |  |  |  |  |  |  |
| *Cupido decoloratus* | EZROM193-08 |  | KJ671885.1 |  |  |  |  |
| *Cupido lorquinii* | EZSPN684-09 |  |  |  |  |  |  |
| *Cupido minimus* | EZHBA678-07 | GQ128845.1 | GQ128636.1 |  |  | GQ128563.1 |  |
| *Cupido osiris* | GBGL0856-06 |  | KJ671887.1 |  |  |  |  |
| *Cyaniris semiargus* | GBGL0855-06 | JX093412.1 | AY496847.1 |  |  | JX093259.1 |  |
| *Erebia aethiopella* | GBMAB1057-15 | KR139118.1 |  |  | KR139008.1 |  |  |

**Table S4**. Continued.

| **Species** | **COI** | **WGL** | **EF1A** | **RPS5** | **GAPDH** | **CAD** | **IDH** |
| --- | --- | --- | --- | --- | --- | --- | --- |
| *Erebia aethiops* | EZHBA304-07 | KR139132.1 |  | KR138884.1 | KR139003.1 |  |  |
| *Erebia alberganus* | GBMAB1064-15 | KR231856.1 |  | KR138913.1 | KR139028.1 |  |  |
| *Erebia arvernensis* | EZSPM093-09 |  |  |  |  |  |  |
| *Erebia calcaria* | GBGL36919-19 | LC340870.1 |  |  |  |  |  |
| *Erebia cassioides* | PHLAA339-09 | KR139120.1 |  | KR138892.1 | KR139010.1 |  |  |
| *Erebia christi* | GBGL36927-19 | LC340957.1 |  |  |  |  |  |
| *Erebia claudina* | EULEP3784-16 | LC340941.1 |  |  |  |  |  |
| *Erebia disa* | GUPPY154-17 | KR139165.1 |  | KR138971.1 | KR139075.1 |  |  |
| *Erebia embla* | EZHBA868-07 | LC340927.1 |  |  |  |  |  |
| *Erebia epiphron* | GBLN0696-06 | DQ338638.1 | DQ338921.1 | KR138888.1 | KR139006.1 |  |  |
| *Erebia epistygne* | EZSPN145-09 | KR139099.1 |  | KR138872.1 | KR138991.1 |  |  |
| *Erebia eriphyle* | EULEP2794-15 | LC340955.1 |  | KR138881.1 | KR139000.1 |  |  |
| *Erebia euryale* | EZROM578-08 | LC340868.1 |  | KR138963.1 | KR139068.1 | KU577141.1 | KU577229.1 |
| *Erebia flavofasciata* | LEPPA1273-17 | LC340887.1 |  | KR138899.1 |  |  |  |
| *Erebia gorge* | EZROM326-08 | KR139137.1 |  | KR138911.1 | KR138986.1 |  |  |
| *Erebia gorgone* | EZSPM197-09 | LC340892.1 |  |  |  |  |  |
| *Erebia hispania* | GBMAB1127-15 | KR139125.1 |  | KR138898.1 | KR139015.1 |  |  |
| *Erebia lefebvrei* | GBMAB1138-15 | KR139084.1 |  | KR138857.1 | KR138976.1 |  |  |
| *Erebia ligea* | GBLN0697-06 | DQ338639.1 | DQ338922.1 | KR138904.1 | KR138989.1 |  |  |
| *Erebia manto* | EZROM156-08 | KR139155.1 |  | KR138968.1 | KR139002.1 |  |  |
| *Erebia medusa* | EZROM581-08 | KR139139.1 |  | KR138914.1 |  |  |  |

**Table S4**. Continued.

| **Species** | **COI** | **WGL** | **EF1A** | **RPS5** | **GAPDH** | **CAD** | **IDH** |
| --- | --- | --- | --- | --- | --- | --- | --- |
| *Erebia melampus* | ODOPE674-11 | KR231871.1 |  | KR138882.1 | KR139001.1 |  |  |
| *Erebia melas* † | EZROM165-08 | KR231873.1 |  | KR138942.1 | KR139052.1 |  |  |
| *Erebia meolans* | DMAZ119-09 | KR139112.1 |  | KR138886.1 |  |  |  |
| *Erebia mnestra* | PHLAB378-10 | LC340846.1 |  |  |  |  |  |
| *Erebia montana* | PHLSA393-11 | KR139123.1 |  | KR138896.1 | KR139013.1 |  |  |
| *Erebia neleus* | EZROM148-08 |  |  |  |  |  |  |
| *Erebia neoridas* | EZSPN180-09 | KR231876.1 |  | KR138930.1 | KR139044.1 |  |  |
| *Erebia nivalis* | GWOSU031-11 | LC340848.1 |  |  |  |  |  |
| *Erebia oeme* | GBLN0698-06 | DQ338640.1 | DQ338923.1 | EU141375.1 | EU141479.1 | EU141296.1 | EU141532.1 |
| *Erebia orientalis* | EULEP907-15 | LC340959.1 |  |  | KR139027.1 |  |  |
| *Erebia ottomana* | PHLAF347-11 | KR231878.1 |  | KR138956.1 | KR139064.1 |  |  |
| *Erebia palarica* | GBLN0145-06 | AY090145.1 | AY090178.2 | GQ357551.1 | GQ357422.1 |  |  |
| *Erebia pandrose* | EZHBA589-07 | LC340967.1 |  | KR138853.1 | KR138972.1 |  |  |
| *Erebia pharte* | LEATJ1259-16 | KR231881.1 |  | KR138928.1 | KR139057.1 |  |  |
| *Erebia pluto* | PHLAA302-09 | KR139089.1 |  | KR138862.1 | KR138981.1 |  |  |
| *Erebia polaris* | LEFIF127-10 |  |  |  |  |  |  |
| *Erebia pronoe* | EZROM628-08 | KR139138.1 |  | KR138912.1 | KR139026.1 |  |  |
| *Erebia rhodopensis* | GBGL37181-19 | KR139133.1 |  |  |  |  |  |
| *Erebia scipio* | GBGL37189-19 | LC340949.1 |  |  |  |  |  |
| *Erebia sthennyo* | EZSPM561-10 | DQ338641.1 | DQ338924.1 | KR138885.1 | KR139005.1 |  |  |
| *Erebia stiria* | LEATG428-14 |  |  |  |  |  |  |

**Table S4**. Continued.

| **Species** | **COI** | **WGL** | **EF1A** | **RPS5** | **GAPDH** | **CAD** | **IDH** |
| --- | --- | --- | --- | --- | --- | --- | --- |
| *Erebia styx* | GBMAB1220-15 | LC340942.1 |  | KR138925.1 | KR139040.1 |  |  |
| *Erebia sudetica* | EZROM172-08 | LC340858.1 |  | KR138960.1 |  |  |  |
| *Erebia triaria* | GBLN0700-06 | LC340886.1 | DQ338925.1 | KR138859.1 | KR138978.1 |  |  |
| *Erebia tyndarus* | GBMAB1237-15 | KR139128.1 |  | KR138901.1 | KR139016.1 |  |  |
| *Erebia zapateri* | EZSPM541-10 | LC340896.1 |  |  |  |  |  |
| *Erynnis marloyi* | IRANB189-08 |  |  |  |  |  |  |
| *Erynnis tages* | EZHBA474-07 | EU442875.1 |  |  |  |  |  |
| *Euchloe ausonia* | LOWA791-06 |  |  |  |  |  |  |
| *Euchloe bazae* | EZSPN161-09 |  |  |  |  |  |  |
| *Euchloe belemia* | EZSPN318-09 |  |  |  |  |  |  |
| *Euchloe crameri* | EZSPN099-09 |  |  |  |  |  |  |
| *Euchloe insularis* | GBGL6351-09 |  |  |  |  |  |  |
| *Euchloe penia* | GBGL5082-08 |  |  |  |  |  |  |
| *Euchloe simplonia* | EZSPN034-09 |  |  |  |  |  |  |
| *Euchloe tagis* | EZSPN343-09 |  |  |  |  |  |  |
| *Euphydryas aurinia* | GBLN0040-06 | AY788504.1 | AY788743.1 | HQ424731.1 |  |  |  |
| *Euphydryas cynthia* | GWOTF678-12 |  |  |  |  |  |  |
| *Euphydryas desfontainii* | GBLN0159-06 | AY090159.1 | AY090193.1 |  |  |  |  |
| *Euphydryas iduna* | LEFIA735-10 |  |  |  |  |  |  |
| *Euphydryas intermedia* | EZHBA567-07 | KX824735.1 | KX824703.1 |  |  |  |  |
| *Euphydryas maturna* | LOWA187-06 |  |  |  |  |  |  |

**Table S4**. Continued.

| **Species** | **COI** | **WGL** | **EF1A** | **RPS5** | **GAPDH** | **CAD** | **IDH** |
| --- | --- | --- | --- | --- | --- | --- | --- |
| *Favonius quercus* | LOWA765-06 | GU829582.1 | GU829029.1 | GU830694.1 |  | JN204939.1 | GU830092.1 |
| *Gegenes nostrodamus* | VNMB335-08 |  |  |  |  |  |  |
| *Gegenes pumilio* | GBGL30143-19 |  |  |  |  |  |  |
| *Glaucopsyche alexis* | GBGL35040-19 |  | AY675366.1 |  |  |  |  |
| *Glaucopsyche melanops* | EZSPN086-09 | HQ918000.1 | JF271992.1 |  |  |  |  |
| *Gonepteryx cleopatra* | GBGLP389-14 | KM046578.1 |  | KM046630.1 | KM046870.1 | KM046524.1 | KM046761.1 |
| *Gonepteryx farinosa* | IRANB296-08 |  |  |  |  |  |  |
| *Gonepteryx rhamni* | GBGL6442-09 |  | AY870568.1 |  |  |  |  |
| *Hamearis lucina* | GBMIN87811-17 | KT285990.1 | DQ018920.1 | EU141425.1 |  | KT286300.1 | EU141584.1 |
| *Hesperia comma* | GUPPY112-17 | AY700706.1 |  |  |  |  |  |
| *Heteropterus morpheus* | EZROM208-08 | KY019979.1 |  |  | KY027571.1 | KY045614.1 | KY027818.1 |
| *Hipparchia aristaeus* | WMB036-11 |  |  |  |  |  |  |
| *Hipparchia blachieri* | WMB2750-13 |  |  |  |  |  |  |
| *Hipparchia cretica* | EZROM216-08 |  |  |  |  |  |  |
| *Hipparchia fagi* | EZROM319-08 |  |  |  |  |  |  |
| *Hipparchia fatua* | EZROM603-08 |  |  |  |  |  |  |
| *Hipparchia fidia* | GBLN0661-06 | DQ338731.1 |  |  |  |  |  |
| *Hipparchia hermione* | EZSPN505-09 |  |  |  |  |  |  |
| *Hipparchia leighebi* | HBOK002-08 |  |  |  |  |  |  |
| *Hipparchia neomiris* | WMB865-13 |  |  |  |  |  |  |
| *Hipparchia semele* | GBLN0785-06 | DQ338732.1 | DQ339023.1 |  |  |  |  |

**Table S4**. Continued.

| **Species** | **COI** | **WGL** | **EF1A** | **RPS5** | **GAPDH** | **CAD** | **IDH** |
| --- | --- | --- | --- | --- | --- | --- | --- |
| *Hipparchia senthes* | GBGL37321-19 |  |  |  |  |  |  |
| *Hipparchia statilinus* | GBLN0662-06 | DQ338733.1 | DQ339024.1 | GQ357629.1 | GQ357503.1 |  |  |
| *Hipparchia syriaca* | EZROM1013-08 |  |  |  |  |  |  |
| *Hipparchia volgensis* | EZROM216-08 |  | KY000502.1 |  |  |  |  |
| *Hyponephele lupina* | GBGL37333-19 |  |  |  |  |  |  |
| *Hyponephele lycaon* | LOWA317-06 | KT448714.1 | KT448655.1 |  |  |  |  |
| *Iolana iolas* | LEATG482-14 | HQ918002.1 | AY675367.1 |  |  |  |  |
| *Iphiclides podalirius* | GBGL0203-06 | DQ351129.1 | AF173413.2 |  |  |  |  |
| *Issoria lathonia* | GBLN0826-06 | DQ922822.1 | DQ922886.1 | KY773489.1 | KY773384.1 |  |  |
| *Kirinia climene* | GBLN3137-10 | GQ357318.1 | GQ357252.1 | GQ357514.1 | GQ357386.1 |  |  |
| *Kirinia roxelana* | GBLN0533-06 | DQ176325.1 | DQ338908.1 | GQ357515.1 | GQ357387.1 |  |  |
| *Kretania eurypilus* | GBGL0955-06 | JX093433.1 | JX093298.1 |  |  | JX093265.1 |  |
| *Kretania hesperica* | EZSPN166-09 |  |  |  |  |  |  |
| *Kretania psylorita* | GBMIN81766-17 |  |  |  |  |  |  |
| *Kretania sephirus* | GBGL10089-12 |  |  |  |  |  |  |
| *Kretania trappi* | LEATG565-14 |  |  |  |  |  |  |
| *Laeosopis roboris* | EZSPN478-09 |  |  |  |  |  |  |
| *Lampides boeticus* * | GBGL0030-06 | GQ128924.1 | GQ128712.1 |  |  |  |  |
| *Lasiommata maera* | GBLN0535-06 | DQ176328.1 |  |  |  |  |  |
| *Lasiommata megera* | GBLN0536-06 | AY090146.1 | AY090179.2 | GQ357516.1 | GQ357388.1 |  |  |
| *Lasiommata paramegaera* | WMB847-13 |  |  |  |  |  |  |

**Table S4**. Continued.

| **Species** | **COI** | **WGL** | **EF1A** | **RPS5** | **GAPDH** | **CAD** | **IDH** |
| --- | --- | --- | --- | --- | --- | --- | --- |
| *Lasiommata petropolitana* | GBLN0537-06 | DQ176327.1 |  |  |  |  |  |
| *Leptidea duponcheli* | GBMIN34112-13 | JF513004.1 |  |  |  |  |  |
| *Leptidea juvernica* ^2^ | GBGLP353-14 | JF512968.1 |  |  |  | JF512751.1 |  |
| *Leptidea morsei* | GBGLP359-14 | JF512996.1 | GU372654.1 |  |  | JF512749.1 |  |
| *Leptidea reali* ^2^ | GBGL4924-08 | JF512941.1 |  |  |  | JF512722.1 |  |
| *Leptidea sinapis* ^2^ | GBGL1313-06 | AY954595.1 | AY870573.1 | GU830806.1 | GU829910.1 | GU828319.1 | GU830237.1 |
| *Leptotes pirithous* * | GBGL0825-06 |  | KJ671872.1 |  |  |  |  |
| *Libythea celtis* | GBLN0131-06 | AY090131.1 | AY090164.2 | EU141418.1 | EU141517.1 | EU141340.1 | EU141577.1 |
| *Limenitis camilla* | ABOLB037-15 | EU098268.1 | EF643322.1 | HQ291253.1 | AB976128.1 |  | HQ291221.1 |
| *Limenitis populi* | EZROM247-08 | AB976214.1 | DQ208227.1 | HQ291256.1 | AB976144.1 |  | HQ291227.1 |
| *Limenitis reducta* | GBLN0150-06 | AY090150.1 | AY090183.1 | EU141409.1 | EU141509.1 | EU141331.1 | EU141568.1 |
| *Lopinga achine* | GBLN0534-06 | DQ176329.1 | DQ338910.1 | GQ357517.1 | GQ357389.1 |  |  |
| *Lycaena alciphron* | GBGL31243-19 |  | JN204975.1 | JN205041.1 |  |  | JN204998.1 |
| *Lycaena dispar* | BJUP525-17 |  | GU372655.1 |  |  |  |  |
| *Lycaena helle* | EZHBA653-07 |  |  |  |  |  |  |
| *Lycaena hippothoe* | EZROM258-08 |  |  |  |  |  |  |
| *Lycaena ottomana* | KT581641.1 |  |  |  |  |  |  |
| *Lycaena phlaeas* | GBGL31246-19 |  | FJ490506.1 |  | AB696721.1 |  |  |
| *Lycaena thersamon* | GBGL31248-19 |  |  |  |  |  |  |
| *Lycaena thetis* | GBGL0993-06 |  |  |  |  |  |  |
| *Lycaena tityrus* | GBMIN38811-13 |  | FJ490509.1 |  |  |  |  |

**Table S4**. Continued.

| **Species** | **COI** | **WGL** | **EF1A** | **RPS5** | **GAPDH** | **CAD** | **IDH** |
| --- | --- | --- | --- | --- | --- | --- | --- |
| *Lycaena virgaureae* | GBMIN38813-13 |  | FJ490505.1 |  |  |  |  |
| *Lysandra albicans* | GBGL0837-06 | KF834541.1 |  |  |  | KF834324.1 |  |
| *Lysandra bellargus* | GBGLL813-15 | JX093410.1 | AY496826.1 |  |  | JX093262.1 |  |
| *Lysandra coridon* | LEATG476-14 | GQ128874.1 | GQ128665.1 |  |  | GQ128594.1 |  |
| *Lysandra hispana* | GBGL35247-19 | KF834538.1 |  |  |  | KF834323.1 |  |
| *Maniola jurtina* | GBLN0147-06 | AY090147.1 | AY090180.2 | EU141376.1 | EU141481.1 | EU141298.1 | EU141534.1 |
| *Maniola nurag* | GBLN5406-15 | KP032479.1 | KP032584.1 |  |  |  |  |
| *Melanargia arge* | GBMIN86457-17 | GQ201377.1 |  |  |  |  |  |
| *Melanargia galathea* | GBLN0761-06 | DQ338706.1 | DQ338993.1 | EU528444.1 | EU528398.1 |  |  |
| *Melanargia ines* | VNMB140-08 | GQ201390.1 |  |  |  |  |  |
| *Melanargia lachesis* | GBLN3102-10 | GQ201393.1 | GQ357289.1 | GQ357593.1 | GQ357463.1 |  |  |
| *Melanargia larissa* | VNMB193-08 | GQ201396.1 |  |  |  |  |  |
| *Melanargia occitanica* | VNMB563-08 | GQ201405.1 |  |  |  |  |  |
| *Melanargia russiae* | GBLN0763-06 | DQ338708.1 | DQ338995.1 |  |  |  |  |
| *Melitaea aetherie* | EZSPN451-09 | FJ462165.1 | FJ462290.1 |  |  |  |  |
| *Melitaea arduinna* | GBLN0036-06 | AY788534.1 | AY788774.1 | KJ723580.1 |  | KJ723520.1 |  |
| *Melitaea asteria* | PHLSA404-11 |  | FJ462296.1 |  |  |  |  |
| *Melitaea athalia* | MBMPA106-07 | FJ462171.1 | FJ462297.1 |  |  |  |  |
| *Melitaea aurelia* | MBMPA184-07 | FJ462173.1 | FJ462299.1 |  |  |  |  |
| *Melitaea britomartis* | GBLN0453-06 | AY788535.1 | FJ462302.1 |  |  |  |  |
| *Melitaea cinxia* | GBLN0454-06 | AY788536.1 | AY788776.1 | EU141420.1 | EU141518.1 | EU141342.1 | EU141579.1 |

**Table S4**. Continued.

| **Species** | **COI** | **WGL** | **EF1A** | **RPS5** | **GAPDH** | **CAD** | **IDH** |
| --- | --- | --- | --- | --- | --- | --- | --- |
| *Melitaea deione* | GBLN0455-06 | AY788537.1 | FJ462313.1 |  |  |  |  |
| *Melitaea diamina* | MBMPA077-07 | FJ462189.1 | FJ462317.1 |  |  |  |  |
| *Melitaea didyma* | GBMIN85907-17 | FJ462190.1 | FJ462318.1 |  |  |  |  |
| *Melitaea ornata* ^3^ | GBMIN86007-17 | KJ723608.1 | KJ723626.1 | KJ723584.1 | KJ723545.1 | KJ723522.1 |  |
| *Melitaea parthenoides* | LEASS955-17 | FJ462211.1 | FJ462339.1 |  |  |  |  |
| *Melitaea phoebe* ^3^ | GBMIN86029-17 | FJ462214.1 | FJ462342.1 | KJ723595.1 | KJ723556.1 | KJ723533.1 |  |
| *Melitaea trivia* | GBLN0104-06 | AY788542.1 | FJ462355.1 | KJ723577.1 |  | KJ723518.1 |  |
| *Melitaea varia* | GBLN0106-06 | AY788543.1 | FJ462356.1 |  |  |  |  |
| *Minois dryas* | GBLN0004-06 |  | JX185940.1 |  |  |  |  |
| *Neolysandra coelestina* | GBGL0905-06 | JX093417.1 | JX093303.1 |  |  | JX093258.1 |  |
| *Neptis rivularis* | LOWA170-06 | EU098261.1 | JX185943.1 |  | MG741565.1 |  | MG741507.1 |
| *Neptis sappho* | GBGL10107-12 | MG741934.1 | JX185944.1 |  | AB976163.1 |  | MG741511.1 |
| *Nymphalis antiopa* * | GBLN0268-06 | AY218284.1 | AY218266.1 | FJ639579.1 | FJ639524.1 |  |  |
| *Nymphalis c-album* | GBLN0155-06 | AF412771.1 | AY090188.1 | FJ639551.1 | FJ639499.1 | HQ734879.1 | HQ735006.1 |
| *Nymphalis egea* | GBLN0297-06 | AF412779.1 | AY248825.1 | FJ639562.1 | FJ639507.1 |  |  |
| *Nymphalis polychloros* | GBLN0285-06 | AF412787.1 | AY248813.1 | EU141401.1 | EU141502.1 | EU141323.1 | EU141560.1 |
| *Nymphalis vaualbum* | HBNKB284-07 | KX824733.1 | EF683651.1 |  |  |  |  |
| *Nymphalis xanthomelas* | GBLN0287-06 | AF412783.1 | JX185966.1 | FJ639582.1 | FJ639527.1 |  |  |
| *Ochlodes sylvanus* | EZROM339-08 |  | JN204972.1 | JN205038.1 |  | JN204945.1 | JN204996.1 |
| *Oeneis bore* | GUPPY122-17 | KP888775.1 | LC155661.1 | KP888737.1 | KP888702.1 |  |  |
| *Oeneis glacialis* | PHLAA392-09 | KP888785.1 | LC155690.1 | KP888746.1 | KP888711.1 |  |  |

**Table S4**. Continued.

| **Species** | **COI** | **WGL** | **EF1A** | **RPS5** | **GAPDH** | **CAD** | **IDH** |
| --- | --- | --- | --- | --- | --- | --- | --- |
| *Oeneis jutta* | GBLN0513-06 | DQ018896.1 | DQ018925.1 | GQ357632.1 | GQ357506.1 |  |  |
| *Oeneis norna* | COLFF475-13 | KP888802.1 | LC155630.1 | KP888757.1 | KP888725.1 |  |  |
| *Papilio alexanor* | GBGL0124-06 | GQ268407.1 | AF044821.1 |  |  |  |  |
| *Papilio hospiton* | GBGL0121-06 |  | AF044830.1 |  |  |  |  |
| *Papilio machaon* | GBGL7241-10 | AY569124.1 | AF044819.1 |  |  |  |  |
| *Pararge aegeria* | GBLN0584-06 | DQ176339.1 | DQ338913.1 | EU141372.1 | EU141476.1 | EU141293.1 | EU141529.1 |
| *Parnassius apollo* | GBGL9492-12 | HM213842.1 | EF485050.1 |  |  |  |  |
| *Parnassius mnemosyne* | GBGL5992-09 |  | EF485081.1 |  |  |  |  |
| *Parnassius phoebus* | GBGL3787-07 | FJ756881.1 | AF173412.2 | JN205043.1 |  | JN204950.1 | JN204999.1 |
| *Phengaris alcon* | GBGL35257-19 | HQ918050.1 | AY675357.1 |  | KM517332.1 |  |  |
| *Phengaris arion* | GBMIN13257-13 | HQ918076.1 | AY675355.1 |  | KM517347.1 |  |  |
| *Phengaris nausithous* | GBGLL228-13 | HQ918006.1 | AY675356.1 |  | KM517356.1 |  |  |
| *Phengaris teleius* | GBGLL391-13 | HQ918008.1 | AY675371.1 |  | KM517369.1 |  |  |
| *Pieris brassicae* | GBMIN87405-17 |  | KM669634.1 |  | AY885236.1 |  |  |
| *Pieris bryoniae* | EZHBA217-07 |  |  |  |  |  |  |
| *Pieris ergane* | LOWAB069-07 |  |  |  |  |  |  |
| *Pieris krueperi* | LOWA913-08 |  |  |  |  |  |  |
| *Pieris mannii* | EZROM463-08 |  |  |  |  |  |  |
| *Pieris napi* | GBGL0192-06 | AY569041.1 | AF173401.1 |  |  |  |  |
| *Pieris rapae* | GBGL1329-06 | AF014148.1 | GU372646.1 | JN205036.1 | JN204991.1 |  | JN205008.1 |
| *Plebejus argus* | GBGL35354-19 | GQ128889.1 | AY496828.1 |  |  | GQ128609.1 |  |

**Table S4**. Continued.

| **Species** | **COI** | **WGL** | **EF1A** | **RPS5** | **GAPDH** | **CAD** | **IDH** |
| --- | --- | --- | --- | --- | --- | --- | --- |
| *Plebejus argyrognomon* | GBGL35370-19 | GQ128870.1 | AY496827.1 |  |  | GQ128590.1 |  |
| *Plebejus idas* | LYCAE526-15 | GQ128871.1 | GQ128662.1 |  |  | GQ128591.1 |  |
| *Polyommatus admetus* | GBGL4334-07 |  |  |  |  |  |  |
| *Polyommatus amandus* | GBGL35383-19 | JX093418.1 | AY496832.1 |  |  | JX093245.1 |  |
| *Polyommatus aroaniensis* | GBGL0733-06 |  |  |  |  |  |  |
| *Polyommatus celina* | GBGL8638-12 |  |  |  |  |  |  |
| *Polyommatus damon* | GBGL0998-06 | GQ128841.1 | AY496835.1 |  |  | GQ128559.1 |  |
| *Polyommatus daphnis* | GBGL0761-06 | JX093424.1 | AY496845.1 |  |  | JX093252.1 |  |
| *Polyommatus dolus* | GBGL8640-12 |  | AY496833.1 |  |  |  |  |
| *Polyommatus dorylas* | GBGL0869-06 | JX093420.1 | AY496831.1 |  |  | JX093248.1 |  |
| *Polyommatus eros* | GBGL1003-06 |  | KJ671889.1 |  |  |  |  |
| *Polyommatus escheri* | GBGL0732-06 | JX093425.1 | JX093296.1 |  |  | JX093241.1 |  |
| *Polyommatus fabressei* | GBGL0829-06 |  |  |  |  |  |  |
| *Polyommatus fulgens* | GBGL0840-06 |  |  |  |  |  |  |
| *Polyommatus golgus* | EZSPN168-09 |  |  |  |  |  |  |
| *Polyommatus humedasae* | GBGL1005-06 |  |  |  |  |  |  |
| *Polyommatus icarus* | GBGL6592-09 | GQ128891.1 | AY496846.1 |  |  | GQ128611.1 |  |
| *Polyommatus iphigenia* | GBGL4342-07 |  |  |  |  |  |  |
| *Polyommatus nivescens* | EZSPC1332-10 | JX093421.1 | JX093286.1 |  |  | JX093249.1 |  |
| *Polyommatus orphicus* | GBMIN71191-17 |  |  |  |  |  |  |
| *Polyommatus ripartii* | GBGL0839-06 | JX093429.1 | AY496834.1 |  |  | JX093237.1 |  |

**Table S4**. Continued.

| **Species** | **COI** | **WGL** | **EF1A** | **RPS5** | **GAPDH** | **CAD** | **IDH** |
| --- | --- | --- | --- | --- | --- | --- | --- |
| *Polyommatus thersites* | GBGL0836-06 | JX093436.1 | JX093291.1 |  |  | JX093250.1 |  |
| *Polyommatus virgilia* | HM210162.1 |  |  |  |  |  |  |
| *Pontia callidice* | LEATG067-14 | KM046589.1 | KM669641.1 | KM046660.1 | KM046900.1 | KM046551.1 | KM046785.1 |
| *Pontia chloridice* | LOWA426-06 |  | JX495369.1 |  |  |  |  |
| *Pontia daplidice* * | BJUP258-17 |  | JX495392.1 |  |  |  |  |
| *Pontia edusa* * | EZROM510-08 |  | KM669640.1 |  |  |  |  |
| *Proterebia afra* | GBGL37695-19 | GQ357353.1 | GQ357288.1 | GQ357590.1 | GQ357460.1 |  |  |
| *Pseudochazara amymone* | GBMIN86626-17 |  |  |  |  |  |  |
| *Pseudochazara anthelea* | KU360266.1 |  |  |  |  |  |  |
| *Pseudochazara cingovskii* | GBMIN86629-17 |  |  |  |  |  |  |
| *Pseudochazara geyeri* | GBMIN71228-17 |  |  |  |  |  |  |
| *Pseudochazara graeca* † | GBMIN86632-17 |  |  |  |  |  |  |
| *Pseudochazara orestes* | GBMIN86637-17 |  |  |  |  |  |  |
| *Pseudochazara tisiphone* | GBMIN86643-17 |  |  |  |  |  |  |
| *Pseudochazara williamsi* | EZSPC1431-10 |  |  |  |  |  |  |
| *Pyrgus alveus* | FBLMW350-10 |  |  |  |  |  |  |
| *Pyrgus andromedae* | EZRMN363-08 |  |  |  |  |  |  |
| *Pyrgus armoricanus* | EZROM522-08 |  |  |  |  |  |  |
| *Pyrgus bellieri* † | EZSPC006-09 |  |  |  |  |  |  |
| *Pyrgus cacaliae* | ABOLB022-15 |  |  |  |  |  |  |
| *Pyrgus carlinae* | LEPAA381-16 |  |  |  |  |  |  |

**Table S4**. Continued.

| **Species** | **COI** | **WGL** | **EF1A** | **RPS5** | **GAPDH** | **CAD** | **IDH** |
| --- | --- | --- | --- | --- | --- | --- | --- |
| *Pyrgus carthami* | EZSPM350-09 |  |  |  |  |  |  |
| *Pyrgus centaureae* | HBNK721-07 |  |  |  |  |  |  |
| *Pyrgus cinarae* | EZSPC612-09 |  |  |  |  |  |  |
| *Pyrgus cirsii* | EZSPC111-09 |  |  |  |  |  |  |
| *Pyrgus malvae* | GWOR3938-09 | GU829483.1 | GU828922.1 | GU830600.1 | GU829744.1 | GU828085.1 | GU829972.1 |
| *Pyrgus onopordi* | EZSPN102-09 |  |  |  |  |  |  |
| *Pyrgus serratulae* | GBGL36400-19 |  |  |  |  |  |  |
| *Pyrgus sidae* | EZSPN022-09 |  |  |  |  |  |  |
| *Pyrgus warrenensis* | EZSPC024-09 |  |  |  |  |  |  |
| *Pyronia bathseba* | GBLN0759-06 |  |  |  |  |  |  |
| *Pyronia cecilia* | GBLN0760-06 |  |  |  | GQ357461.1 |  |  |
| *Pyronia tithonus* | EZROM534-08 |  |  |  |  |  |  |
| *Satyrium acaciae* | EZROM536-08 |  | KM211589.1 |  |  |  |  |
| *Satyrium esculi* | GBGL0827-06 |  |  |  |  |  |  |
| *Satyrium ilicis* | OXB1094-15 |  |  |  |  |  |  |
| *Satyrium pruni* | EZROM542-08 |  | GU372631.1 |  |  |  |  |
| *Satyrium spini* | EZROM324-08 |  | KM211587.1 |  |  |  |  |
| *Satyrium w-album* | EULEP203-14 |  |  |  |  |  |  |
| *Satyrus actaea* | GBLN2808-10 | DQ338738.1 | DQ339029.1 | EU528463.1 | EU528412.1 | GQ864709.1 | GQ865146.1 |
| *Satyrus ferula* | GBLN0789-06 | DQ338739.1 | DQ339030.1 | KM200281.1 | KM200255.1 |  |  |
| *Scolitantides abencerragus* | EZSPN385-09 | MH249110.1 | MH235240.1 |  |  |  |  |

**Table S4**. Continued.

| **Species** | **COI** | **WGL** | **EF1A** | **RPS5** | **GAPDH** | **CAD** | **IDH** |
| --- | --- | --- | --- | --- | --- | --- | --- |
| *Scolitantides barbagiae* | WMB317-11 | MH249099.1 | MH235236.1 |  |  |  |  |
| *Scolitantides baton* | EZSPN231-09 | MH249093.1 | AY675369.1 |  |  |  |  |
| *Scolitantides bavius* | EULEP3114-15 | HQ918017.1 | AY675358.1 |  |  |  |  |
| *Scolitantides orion* | EZRMN273-08 | HQ918021.1 | AY675394.1 |  |  |  |  |
| *Scolitantides panoptes* | EZSPN140-09 | HQ918020.1 | JF271997.1 |  |  |  |  |
| *Scolitantides vicrama* | GBGL0920-06 | MH249094.1 | AY675374.1 |  |  |  |  |
| *Spialia orbifer* | GBGL36402-19 | KU905648.1 |  |  |  |  |  |
| *Spialia phlomidis* | GBGL36403-19 |  |  |  |  |  |  |
| *Spialia sertorius* | EULEP011-14 | EU363983.1 | EU364176.1 | KY028701.1 | KY027678.1 | KY045722.1 | KY027920.1 |
| *Syrichtus cribrellum* | EZHBA437-07 |  |  |  |  |  |  |
| *Syrichtus proto* † | EZSPN185-09 |  |  |  |  |  |  |
| *Syrichtus tessellum* † | EZROM434-08 |  | JX495379.1 |  |  |  |  |
| *Tarucus balkanicus* | MABUT061-10 |  | KJ671874.1 |  |  |  |  |
| *Tarucus theophrastus* | GBGL0851-06 |  |  |  |  |  |  |
| *Thecla betulae* | EZHBA261-07 |  | GU372690.1 |  |  |  |  |
| *Thymelicus acteon* | IRANB190-08 |  |  |  |  |  |  |
| *Thymelicus hyrax* | IRANB192-08 |  |  |  |  |  |  |
| *Thymelicus lineola* | NGSFT3895-16 | JN204920.1 | JN204978.1 | JN205029.1 | JN204987.1 | JN204935.1 | JN205002.1 |
| *Thymelicus sylvestris* | LOWAB078-07 |  |  |  |  |  |  |
| *Tomares ballus* | TOMAR126-09 | KT286081.1 | KT286264.1 |  |  | KT286378.1 |  |
| *Tomares nogelii* | TOMAR122-09 |  |  |  |  |  |  |

**Table S4**. Continued.

| **Species** | **COI** | **WGL** | **EF1A** | **RPS5** | **GAPDH** | **CAD** | **IDH** |
| --- | --- | --- | --- | --- | --- | --- | --- |
| *Turanana taygetica* | IRANB369-08 |  |  |  |  |  |  |
| *Vanessa atalanta* * | GBLN0154-06 | AF246542.1 | AY090187.1 | GQ865508.1 | GQ865045.1 | GQ864722.1 | GQ865155.1 |
| *Vanessa cardui* * | GBLN0279-06 | AF412770.1 | AY248807.1 | HQ735031.1 | HQ734956.1 | HQ734873.1 | HQ734993.1 |
| *Vanessa virginiensis* * | GBLN0492-06 | AF412776.1 | AY248808.1 | HQ735033.1 | HQ734958.1 | KJ648964.1 | HQ734995.1 |
| *Zegris eupheme* | EZHBA680-07 | KM046596.1 | AY870559.1 | KM046667.1 | KM046910.1 | KM046561.1 | KM046795.1 |
| *Zerynthia cassandra* | WMB622-11 |  |  |  |  |  |  |
| *Zerynthia cerisyi* | GBGL0199-06 | DQ351146.1 | AF173409.2 |  |  |  |  |
| *Zerynthia cretica* | GBGL4838-08 | DQ351148.1 | DQ351121.1 |  |  |  |  |
| *Zerynthia polyxena* | GBGL4834-08 | DQ351145.1 | DQ351119.1 |  |  |  |  |
| *Zerynthia rumina* | GBGL0200-06 | DQ351144.1 | AF173410.2 |  |  |  |  |
| *Zizeeria knysna* | GBGL0849-06 |  | KJ671876.1 |  |  |  |  |

* Migrant species not considered for analyses.
† Disregarded due to an insufficient number of occupied cells to calculate their climate niche breadths.
^1^ Merged and treated as *Coenonympha gardetta*.
^2^ Merged and treated as *Leptidea sinapis*.
^3^ Merged and treated as *Melitaea phoebe*.

**Table S5.** Best-fitting partitioning scheme for the butterfly sequence data set determined by Partitionfinder. This scheme was applied for tree estimation in BEAST.

| **Partition** | **Best Model** | **Subset Partitions** |
| --- | --- | --- |
| 1 | GTR+I+G | WGL_pos1 |
| 2 | SYM+I+G | WGL_pos2 |
| 3 | GTR+I+G | GAPDH_pos1, WGL_pos3, EF1A_pos3 |
| 4 | GTR+I+G | CAD_pos3, GAPDH_pos2, IDH_pos3, EF1A_pos1, RPS5_pos1 |
| 5 | SYM+I+G | EF1A _pos2 |
| 6 | GTR+I+G | GAPDH_pos3, RPS5_pos2 |
| 7 | GTR+I+G | CAD_pos2, IDH_pos2, RPS5_pos3 |
| 8 | GTR+I+G | CAD_pos1, IDH_pos1 |
| 9 | GTR+G | COI_pos1 |
| 10 | SYM+I+G | COI_pos2 |
| 11 | GTR+I+G | COI_pos3 |

**Table S6.** Calibration points used for time-calibration of the butterfly phylogeny with detailed prior settings as applied in BEAST.

| **Clade** | **Type of prior** | **Mean** | **Sigma** | **Offset** | **Comment** |
| --- | --- | --- | --- | --- | --- |
| Hesperiidae | normal | 51.9 | 5 | 0 |  |
| Lycaenidae | normal | 56.45 | 5 | 0 |  |
| Nymphalidae | normal | 82.04 | 5 | 0 |  |
| Papilionidae | normal | 58.91 | 5 | 0 |  |
| Pieridae | normal | 76.9 | 5 | 0 |  |
| root | log normal | 12 | 0.5 | 95 | mean in real space |

**Table S7.** Correlations among predictor variables. Values represent Spearman’s *ρ* for correlations including the rank-based dispersal tendency and Pearson’s *r* otherwise.

| **Variable** | **Climate niche breadth** | **Diet niche breadth** | **Habitat niche breadth** | **Dispersal tendency** |
| --- | --- | --- | --- | --- |
| Diet niche breadth | 0.49 |  |  |  |
| Habitat niche breadth | 0.33 | 0.22 |  |  |
| Dispersal tendency | 0.40 | 0.27 | 0.33 |  |
| Wingspan | 0.08 | 0.00 | −0.02 | 0.11 |

**
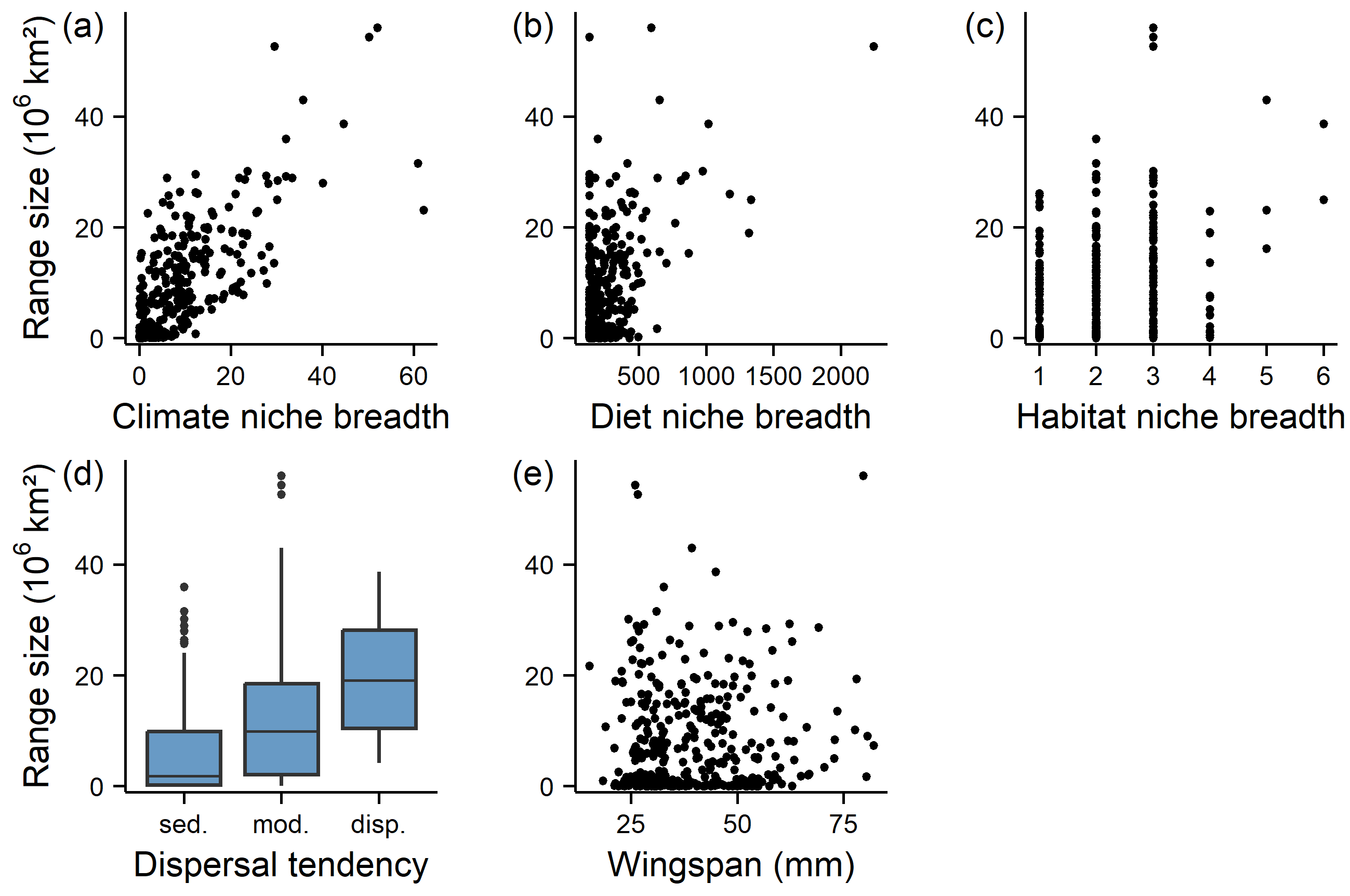
**

**Figure S1.** Relationships between the range sizes of 369 European-centred butterfly species and the five predictor variables describing niche breadths (a – c) and dispersal ability (d – e). Climate niche breadth, diet niche breadth and habitat niche breadth represent hypervolumes derived from a PCA based on 19 bioclimatic variables, the phylogenetic diversity of host plant genera and the number of broad habitat types used for reproduction, respectively. Dispersal tendency (sed., sedentary; mod., moderately mobile; disp., dispersive) was determined by expert assessment, based on Bink (1992) and Settele et al. (1999).

**Sample size and climate niche breadth:**

There are numerous ways to measure the climate niche breadths of species and deal with the issue of varying sample sizes. To assess the robustness of the results we present in the main text, we repeated the analyses using different approaches of climate niche breadth calculation and correction for sample size:

(1) There are simpler metrics available for measuring a species’ climate niche breadth, e.g., using minima and maxima. However, those are heavily influenced by outliers and we consequently dismissed this approach. Yet, in addition to climate niche breadth calculated as the hypervolume derived from a PCA based on 19 bioclimatic variables, we did calculate a simpler metric. Thereby, for every species we extracted the ranges of their principal component scores (excluding the bottom/top 2.5% outliers) along the first four principal components of this PCA. These four ranges were then multiplied to obtain the species climate niche breadth.

(2) We further included the number of observations in GBIF (from which climate niche breadth was calculated) as an additional predictor in our models, alongside our original hypervolume-based climate niche breadth metric. However, climate niche breadth and the number of observations were highly correlated (*r* > 0.7) and should therefore not be used in the same model. Here, we present the results regardless.

(3) Finally, we regressed climate niche breadth against the number of observations in GBIF and then used the residuals of this regression as a climate niche variable, in an attempt to remove the effect of sample size on climate niche breadth.

These alternative approaches produce qualitatively similar results compared to our initial approach (Table S8). We are therefore confident that the results we present in the main text are robust.

**Table S8.** Importance of predictor variables in explaining range size of 369 European-centred butterfly species derived from PGLS models. Values represent the partial R² values derived from comparisons of a full model including all predictors to a set of models lacking the respective predictor. Values for phylogeny were obtained by comparing the full model to an equivalent OLS full model. The value for the full PGLS model represents the total R² value. Partial R² values for the constrained models are given as mean (min – max) across ten replicated model fits. The results for the ‘Unconstrained’ and ‘Constrained’ models are those already presented in the main text, while ‘Simple’, ‘Occurrences’ and ‘Residual’ refer to models calculated using a simpler climate niche breadth metric; models including the number of GBIF observations as a predictor; and models using the residuals of a regression of climate niche breadth against number of GBIF observations as climate niche breadth variable, respectively.

| **Model component** | **Unconstrained** | **Constrained** | **Simple** | **Occurrences** | **Residual** |
| --- | --- | --- | --- | --- | --- |
| Full model | 0.59 | 0.48 (0.46 – 0.49) | 0.50 | 0.59 | 0.43 |
| Climate niche breadth | 0.38 | 0.22 (0.18 – 0.24) | 0.25 | 0.35 | 0.13 |
| Diet niche breadth | 0.00 | 0.04 (0.03 – 0.05) | 0.04 | 0.00 | 0.06 |
| Habitat niche breadth | 0.04 | 0.07 (0.07 – 0.08) | 0.07 | 0.04 | 0.07 |
| Dispersal tendency | 0.00 | 0.00 (0.00 – 0.00) | 0.00 | 0.00 | 0.01 |
| Wingspan | 0.00 | 0.00 (0.00 – 0.00) | 0.00 | 0.00 | 0.00 |
| No. of occupied cells |  |  |  | 0.01 |  |
| Phylogeny | 0.07 | 0.07 (0.06 – 0.09) | 0.07 | 0.06 | 0.08 |

**Alternative habitat niche breadth:**

To test whether our results are robust to the approach chosen for calculating habitat niche breadth we calculated another version of the habitat niche breadth variable using an approach conceptually more similar to the calculation of climate niche breadth (i.e., utilising GBIF occurrence records).

We considered GBIF records located in Europe, for which the CORINE land cover data (https://land.copernicus.eu/pan-european/corine-land-cover), a standardised and quality-checked, satellite-based product is available at a 100 m resolution for the year 2018.

The CORINE land cover data includes 44 different land cover classes, which we grouped into the same seven habitat types we defined for our original habitat niche breadth variable. For each species we subsequently extracted the habitat types at the locations of their GBIF occurrence records. We then considered the number of different habitats a species occurred in as the species’ habitat niche breadth. For 17 species we could not calculate this metric, because no GBIF occurrence records were available for the area covered by the CORINE land cover data. All the following statements and results therefore refer to a reduced set of 352 species (compared to the 369 species considered in the original analyses).

Compared to the original habitat niche breadth (as presented in the main text), this new version of the habitat niche breadth was lower for 14, remained the same for 30 and was higher for 308 species (for 271 of which it at least doubled; Figure S1). We consider this a systematic overestimation of the species habitat niche breadth, most likely due to spatial inaccuracies in the land cover data, but especially the occurrence data.


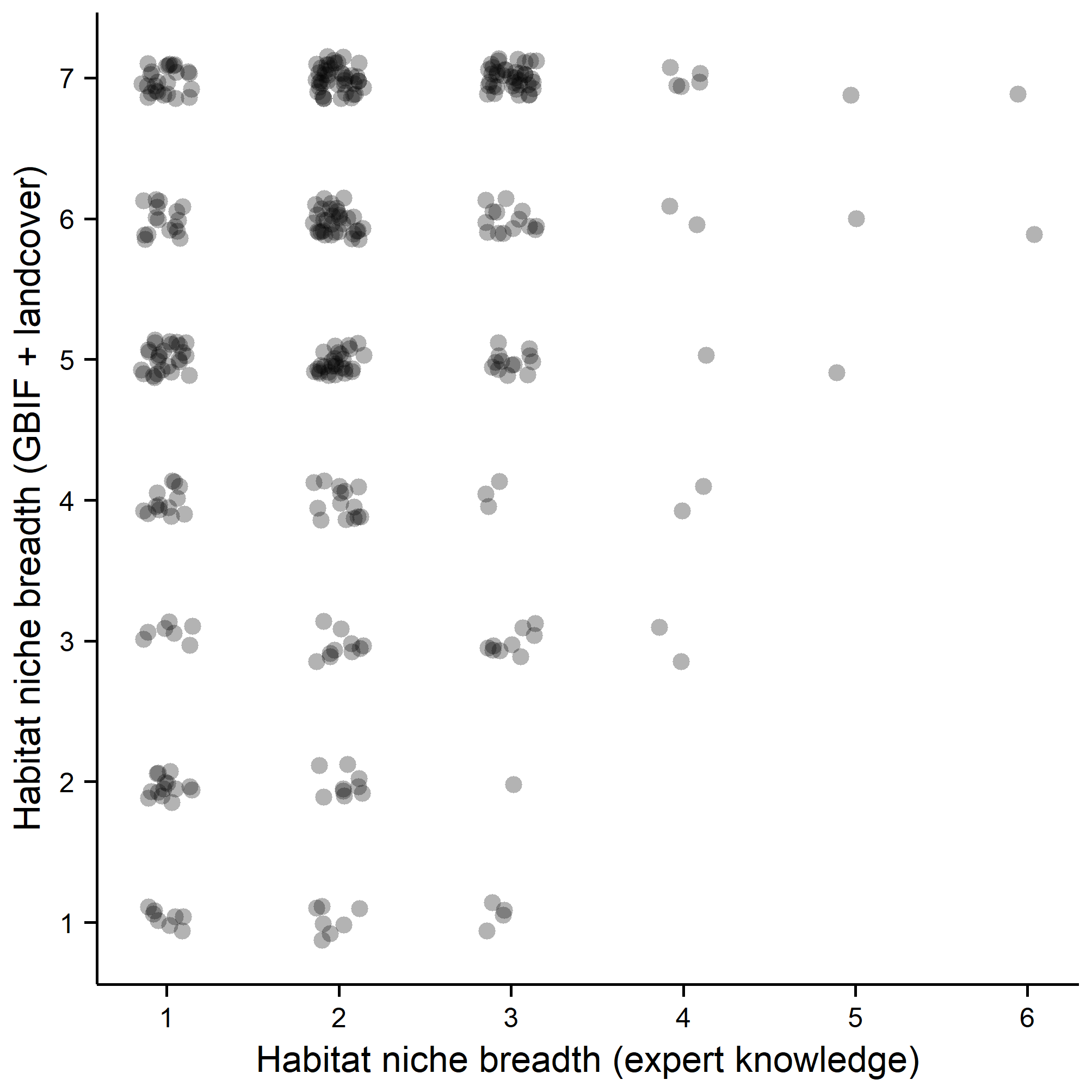


**Figure S1.** The habitat niche breadth derived from GBIF occurrence records and CORINE land cover data, plotted against the original habitat niche breadth which used an expert knowledge-based approach. Please note that while both variables can only take on integer values, we jittered the points to better visualise the distribution of the data.

We also recalculated the PGLS models using this version of the habitat niche breadth variable. The importance of the various predictors in explaining the range sizes of European-centred butterflies remained qualitatively the same (compare Table S9 [below; results using the new variable] and Table 1 [main text; results using the original variable]). While habitat niche breadth was a significant predictor of range size in the unconstrained model when using the original variable, it was a non-significant predictor when using the new version (compare Table S10 below and Table 2 in the main text). In summary, the results remain largely the same. If anything, the importance of habitat niche breadth was reduced when using a habitat niche breadth derived from GBIF occurrence data and CORINE land cover data.

**Table S9.** Importance of predictor variables in explaining range sizes of 352 European-centred butterfly species derived from PGLS models, using a different approach to calculating the species habitat niche breadths (GBIF occurrence records + CORINE landcover). Values represent the partial R² values derived from comparisons of a full model including all predictors to a set of models lacking the respective predictor. Values for the phylogeny were obtained by comparing the full model to an equivalent OLS full model. The value for the full PGLS model represents the total R² value. For the unconstrained models, climate niche breadth was calculated based on the full set of occupied cells, while the replicates of the climate niche breadth for the ten replicated model fits of the constrained models were derived by randomly selecting a maximum of 50 occupied cells per species. Partial R² values for the constrained models are given as mean (min – max) across ten replicated model fits.

| **Model component** | **Unconstrained** | **Constrained** |
| --- | --- | --- |
|  | **R²** | **R²** |
| Full model | 0.62 | 0.55 (0.53 – 0.56) |
| Climate niche breadth | 0.35 | 0.22 (0.19 – 0.24) |
| Diet niche breadth | 0.00 | 0.05 (0.04 – 0.07) |
| Habitat niche breadth | 0.00 | 0.08 (0.07 – 0.08) |
| Dispersal tendency | 0.00 | 0.02 (0.01 – 0.02) |
| Wingspan | 0.00 | 0.00 (0.00 – 0.00) |
| Phylogeny | 0.15 | 0.19 (0.17 – 0.20) |

**Table S10.** Summary of the full PGLS models relating three niche dimensions and two dispersal indicators to the range sizes of 352 European-centred butterfly species using a different approach to calculating the species habitat niche breadths (GBIF occurrence records + CORINE landcover). Estimates for the constrained models represent the means over these ten model fits. Corresponding standard errors were averaged according to Burnham and Anderson (2004; equation 4). The average p-values for the ten replicated model fits were then calculated based on t-values derived from these averaged estimates and standard errors. S.E., standard error.

| **Model component** | **Unconstrained** | | | **Constrained** | | |
| --- | --- | --- | --- | --- | --- | --- |
|  | **Estimate ± S.E.** | **t-value** | **p-value** | **Estimate ± S.E.** | **t-value** | **p-value** |
| Climate niche breadth | 1.54 ± 0.10 | 14.75 | **< 0.001** | 1.01 ± 0.09 | 10.82 | **< 0.001** |
| Diet niche breadth | 0.09 ± 0.10 | 0.92 | 0.357 | 0.45 ± 0.10 | 4.29 | **< 0.001** |
| Habitat niche breadth | 0.05 ± 0.09 | 0.51 | 0.612 | 0.46 ± 0.09 | 5.12 | **< 0.001** |
| Dispersal tendency | 0.15 ± 0.26 | 0.59 | 0.558 | 0.65 ± 0.30 | 2.22 | 0.027 |
| Wingspan | −0.09 ± 0.10 | −0.96 | 0.336 | −0.01 ± 0.11 | −0.08 | 0.936 |

Note: significant effects are shown in bold.
